# Investigating the Function of Human Jumping Translocation Breakpoint Protein (hJTB) and Its Interacting Partners through In-Solution Proteomics of MCF7 Cells

**DOI:** 10.3390/molecules27238301

**Published:** 2022-11-28

**Authors:** Madhuri Jayathirtha, Danielle Whitham, Shelby Alwine, Mary Donnelly, Anca-Narcisa Neagu, Costel C. Darie

**Affiliations:** 1 Biochemistry & Proteomics Laboratories, Department of Chemistry and Biomolecular Science, Clarkson University, Potsdam, NY 13699-5810, USA; 2 Laboratory of Animal Histology, Faculty of Biology, “AlexandruIoanCuza” University of Iasi, Carol I bvd. No. 20A, 700505 Iasi, Romania

**Keywords:** breast cancer, jumping translocation breakpoint (JTB) protein, in-solution proteomics, MCF7, EMT

## Abstract

Human jumping translocation breakpoint (hJTB) gene is located on chromosome 1q21 and is involved in unbalanced translocation in many types of cancer. JTB protein is ubiquitously present in normal cells but it is found to be overexpressed or downregulated in various types of cancer cells, where this protein and its isoforms promote mitochondrial dysfunction, resistance to apoptosis, genomic instability, proliferation, invasion and metastasis. Hence, JTB could be a tumor biomarker for different types of cancer, such as breast cancer (BC), and could be used as a drug target for therapy. However, the functions of the protein or the pathways through which it increases cell proliferation and invasiveness of cancer cells are not well-known. Therefore, we aim to investigate the functions of JTB by using in-solution digestion-based cellular proteomics of control and upregulated and downregulated JTB protein in MCF7 breast cancer cell line, taking account that in-solution digestion-based proteomics experiments are complementary to the initial in-gel based ones. Proteomics analysis allows investigation of protein dysregulation patterns that indicate the function of the protein and its interacting partners, as well as the pathways and biological processes through which it functions. We concluded that JTB dysregulation increases the epithelial-mesenchymal transition (EMT) potential and cell proliferation, harnessing cytoskeleton organization, apical junctional complex, metabolic reprogramming, and cellular proteostasis. Deregulated JTB expression was found to be associated with several proteins involved in mitochondrial organization and function, oxidative stress (OS), apoptosis, and interferon alpha and gamma signaling. Consistent and complementary to our previous results emerged by using in-gel based proteomics of transfected MCF7 cells, JTB-related proteins that are overexpressed in this experiment suggest the development of a more aggressive phenotype and behavior for this luminal type A non-invasive/poor-invasive human BC cell line that does not usually migrate or invade compared with the highly metastatic MDA-MB-231 cells. This more aggressive phenotype of MCF7 cells related to JTB dysregulation and detected by both in-gel and in-solution proteomics could be promoted by synergistic upregulation of EMT, Mitotic spindle and Fatty acid metabolism pathways. However, in both JTB dysregulated conditions, several downregulated JTB-interacting proteins predominantly sustain antitumor activities, attenuating some of the aggressive phenotypical and behavioral traits promoted by the overexpressed JTB-related partners.

## 1. Introduction

Cancer is the leading cause of death in many countries. 2020 cancer statistics show about 2.26 million new cases of breast cancer, 2.20 million new cases of lung cancer, 1.41 million new cases of prostate cancer and 1.14 million new cases of colorectal cancer [1]. There is a rapid increase in the number of cases every year, hence early diagnosis of cancer is important for clinical diagnosis, monitoring toxicity and for the successful treatment of cancers [2]. Biomarkers play a crucial role in early detection of tumors.

In clinic, tumor associated protein-based biomarkers are the most commonly used type of molecular biomarkers [3]. As defined by the World Health Organization, a biomarker is “any substance, structure, or process that can be measured in the body or its products and influence or predict the incidence of outcome or disease” [4]. Also, a biomarker is an indicator of biological or pathogenic process, as well as for assessing the pharmacological responses to therapeutic intervention [2]. The identification of a new biomarker requires the determination of its relevance and validity [5]. Constant research to identify biomarkers that are cost effective and reliable are always in place. Biomarkers help with estimating the risk of cancer and screening for primary cancers, distinguishing between benign and malignant tumors and monitoring the status of the disease [6]. Cancer biomarkers are categorized into predictive biomarkers that predict the risk of developing a cancer, prognostic biomarkers that measure risk of cancer progression or potential response to therapy, and diagnostic biomarkers that indicates the early onset of cancer [3]. Although there are many well-known cancer biomarkers, rapid mutation of genes enables the constant need for new biomarkers. Here, we aim to characterize a new putative biomarker, the JTB protein, which could facilitate early diagnosis and may act as a drug target for the treatment of breast tumors.

JTB is a gene located on human chromosome 1q21 and is involved in unbalanced translocation in many types of cancer such as lung, stomach and colon [7] and most predominantly in breast and prostate cancer [8,9]. JTB protein consists of 146 amino acids and has a molecular weight of 16.4 kDa [10]. It consists of a signal sequence at the N terminus, an extracellular domain rich in cysteine [11], a transmembrane domain that is highly hydrophobic as well an intracellular or a cytoplasmic domain. The JTB protein is ubiquitously present in normal cells but is found to be overexpressed in cancer cells [10]. Hence, this protein could be a tumor biomarker for different types of cancer such as breast, prostate and liver cancers [12] and can be used as a drug target for treatment. However, the function of the protein or the pathways through which it increases cell proliferation is not entirely clear. Hence, we aim to identify the functions of the JTB protein by using in-solution digestion based cellular proteomics that is complementary to the initial gel-based approach previously used in MCF7 BC cells transfected for overexpressed [13] and downregulated JTB condition [14]. Here, we overexpressed and knocked down JTB and looked at the proteomes of the cell for protein dysregulation patterns that indicates the role of the protein and its interacting partners as well as the pathways through which it functions.

MCF7 BC cells were transfected with sense orientation of the hJTB cDNA in a CMV expression vector containing HA, His and FLAG tags to overexpress hJTB and with shRNA plasmid targeting the hJTB mRNA containing an eGFP tag to knockdown the hJTB. The expression levels of upregulated and downregulated JTB conditions were confirmed by Western blotting. The lysates were used for in-solution digestion with trypsin and the digested peptides were analyzed by nano liquid chromatography tandem mass spectrometry (nano LC-MS/MS). Data analysis using Mascot and Scaffold software facilitated the analysis of protein dysregulation patterns. GSEA algorithm was further performed to determine the biological pathways associated with both overexpressed and knockdown conditions of hJTB.

To complement the previously reported JTB proteomics experiments through MS analysis, where we performed in-gel digestion of the upregulated and downregulated JTB samples and their matched controls, an in-solution trypsin digestion of the samples, followed by mass spectrometry-based proteomics analysis was performed, complementarity that was already demonstrated in combinatorial gel electrophoresis [15]. This method is ideal for highly concentrated protein samples; it provides high protein sequence coverage and allows the identification of integral membrane proteins if any in the sample. In addition, the potential problems from in-gel digestion such as poor protein digestion due to gel fixation and insufficient extraction can be avoided in this technique [16].

## 2. Results

We found 18 differentially overexpressed proteins and 14 downregulated proteins compared to control in the overexpressed JTB condition. HSPD1, HSP90AA1, HSPA1A, EEF1A1, RPS14, RPL6, RAN, CAND1, IFITM2, TUBB4A, TUBB2A, TPM3, LAMP2, CREBZF, ENO2, PPIA, PRKCSH and SLC25A5 are overexpressed, while FASN, TPI1, PRDX1, ENO1, SOD1, ACTN4, YWHAQ, CALM1, PCBP1, AHSG, IQGAP2, PDIA4, EEF1A1 and TPD52L2 were found to be downregulated. GSEA was performed for the upregulated JTB condition (Table 1) using H (hallmark gene sets) collection in MSigDB. Analysis of H collection revealed six upregulated pathways, including proteins important for mitotic spindle assembly, epithelial-mesenchymal transition (EMT), fatty acid metabolism (FAM), UV response, interleukin 2 STAT5 signaling, and apoptosis. Two downregulated pathways comprised proteins involved in oxidative phosphorylation (OXPHOS) and apical junction (AJ) pathways.

Similar to overexpressed JTB condition, we found 15 differentially expressed proteins that were upregulated and 16 downregulated compared to control in the knocked-down levels of JTB. PSME1, ENO2, HASPA1A, HSPD1, HSPE1, POTEKP, ACTC1, TUBB, TUBA1A, TMSB10, PARK7, PRDX2, PGK1, GAPDH and PPIA were found to be upregulated and ACTG1, TPI1, HSPA1A, HSPA8, HSPB1, FASN, EEF1A1, ENO2, SOD1, MKI67, CALM1, IFITM2, RPS5, CTNNB1, ISG15, and ANXA2 were found to be downregulated. GSEA was performed for the downregulated JTB condition (Table 2) using H (hallmark gene sets) collection in MSigDB. Analysis of H collection revealed three upregulated pathways, based on proteins involved in complement, interferon gamma response and unfolded protein response (UPR). Five downregulated pathways comprised proteins involved in cholesterol homeostasis, glycolysis, E2F targets, apical junctional complex, hypoxia, and Myc-version 2 pathways.

To study the JTB-related proteins in more detail, we focused on the analysis of protumorigenic (PT) and antitumorigenic (AT) roles of these proteins in correlation with their involvement in cancer-related pathways and biological processes (Table 3 and Table 4). The proteins identified in our study are listed in Supplementary Materials Table S1 and the dysregulated proteins are listed in Supplementary Materials Table S2.

### 2.1. JTB Dysregulation Is Associated with the EMT Process

JTB dysregulation increases the EMT potential and cell proliferation, harnessing cytoskeleton organization, apical junctional complex (AJC), metabolic reprogramming, and cellular proteostasis.

Linked to the alteration of the intracellular skeleton and extracellular matrix (ECM) remodeling, the epithelial-mesenchymal transition (EMT) process facilitates the local invasion in cancer [110]. The EMT pathway has been found as upregulated in our previously published analyses conducted in MCF7 BC cells transfected for JTB overexpression [13] and downregulation, respectively [14], by using SDS-PAGE and nanoLC-MS/MS. We identified then as upregulated proteins related to JTB overexpression filamin A (FLNA), involved in actin cytoskeleton organization and biogenesis, as well as collagen type XI alpha 1 chain (COL11A), and collagen type III alpha1 chain (COL3A1) involved in ECM remodeling [13]. We also identified beta-actin-like protein (ACTBL2), tubulin alpha-4A (TUBA4A), myosin-14 (MYH14), eukaryotic translation elongation factor 1-alpha 1 (EEF1A1), chondroitin sulfate proteoglycan 5 (CSPG5) and clathrin heavy-chain (CLTC) as upregulated EMT-related proteins for downregulated JTB condition [14].

**JTB-related proteinsinvolved in cytoskeletal dynamics and AJC promote EMT**. Taking account that in-solution digestion-based proteomics experiments are complementary to the initial gel-based ones, we identified here more JTB-related proteins that exert several functions and activities involved in cytoskeleton organization and modulation. Thus, we analyzed the biological and pathological functions of actin filaments and microtubules-related proteins identified by using in-solution proteomics. Proteins involved in cytoskeletal dynamics that promote EMT and influence BC metastasis are: TMSB10, TPM3, IQGAP2, ACTC1, ACTG1, ACTN4, TUBA4A, TUBB, TUBA1A, TUBB2A, POTEKP/ACTBL3, and EEF1A1, listed in Table 3 and Table 4. TMSB10 transcriptional factor is involved in cytoskeleton organization and cancer cell migration. It was emphasized as overexpressed in many cancers, such as renal cell carcinoma (RCC), pancreatic cancer, non-small cell lung cancer (NSCLC), papillary thyroid carcinoma [82] and BC [83]. It promotes proliferation, EMT, invasion, migration of cells, and might be used as a serum marker for the diagnosis and potential therapeutic target in BC [83]. TPM3 is also involved in cytoskeleton organization as well as actin alpha cardiac muscle 1 (ACTC1), an overexpressed protein that also promotes the EMT process [95]. There are evidences that demonstrated that TPM3 mRNA is overexpressed in the platelets from patients with metastatic BC and its delivery into BC cells through microvesicles led to an increased migratory behavior and metastasis potency of BC cells [28]. TPM3 mediates EMT and promotes proliferation, invasion and migration of esophageal cancer (EC) cells via metalloproteinase (MMP)2/9 [29]. Overexpression of TPM3 activates SNAIL-mediated EMT, which represses E-cadherin expression and that induces migration and sustains invasion potential of HCC cells during hepatocarcinogenesis [30]. Reduced IQGAP2 expression in upregulated JTB condition could promote EMT by modulating the MEK-ERK and p38 signaling in BC cells [65].

The apical junctional complex (AJC) regulates cell-to-cell adhesion, the paracellular transport, gene transcription, cell proliferation and differentiation, and maintain epithelial cell polarity, acting as tumor suppressor or promoter of cell transformation, migration and metastasis [111]. AJC alteration, as well as the disorganization of the connected actin cytoskeleton, plays an essential role in disturbance of epithelial tissue architecture and cell homeostasis leading to epithelial cancer progression [112]. Three proteins involved in HALLMARK_APICAL_JUNCTION pathway, ACTC1, actin gamma 1 (ACTG1), and actin-binding protein 4 (ACTN4), have been detected as dysregulated in this experiment. The apical junctional complex was identified as downregulated in upregulated JTB condition, according to GSEA algorithm. ACTC1 and ACTG1 are specific isoforms of actin, a key structural protein that makes up the cytoskeleton. Early aberrant expression of actin can be used as a biomarker for malignant transformation, leading to increased migration, cell proliferation and drug resistance [93]. Upregulated ACTC1 was reported in various tumors, such as head and neck cancer, bladder cancer, urothelial cancer, prostate cancer (PCa), NSCLC, BC and glioblastoma (GBM), promoting distant metastasis or multi-drug resistance [93]. There are evidences that indicate the potential role of ACTC1 overexpression in cell motility and cancer cell survival [93]. ACTG1 silencing suppresses the growth of PCa tumors and EMT through MAPK/ERK signaling pathway [113]. Also downregulated in this experiment, ACTN4, that belongs to the family of actin-binding proteins, when overexpressed has been associated with cancer development, aggressiveness, invasion and metastasis, sustaining cell proliferation, motility and EMT [114]. It is predominantly express in the cellular protrusions, such as filopodia and lamellipodia, that encourage the invasive phenotype in cancer cells [115]. However, several proteins that are known as promoters of EMT and that are usually upregulated in cancer cells were found to be downregulated in JTB dysregulated condition. Thus, beta-catenin (CTNNB1), a known EMT-related protein [116], was found as downregulated in this experiment. At this point, it is important to consider that only nuclear accumulation of mutated CTNNB1 was reported to upregulate the EMT process, while the wild type of CTNNB1 showed membrane localization in correlation with a lack of downregulation of claudin-7 and E-cadherin, which could lead to an increasing in cell motility [102]. Downregulated CTNNB1, among other catenin family members, and downregulated expression of cadherin may disrupt the normal cell-cell adhesion machinery in malignant transformed cells that may contribute to enhanced migration, proliferation, invasion and metastasis [109].

According to GSEA algorithm, HALLMARK_MITOTIC_SPINDLE was found as upregulated in upregulated JTB condition as well as the EMT pathway, in accordance with our previous results obtained by SDS-page proteomics [13]. Different tubulin isoforms and their posttranslational modifications (PTMs) emphasized an impact on mitotic spindle assembly and mitosis [117]. We identified as upregulated four tubulins: tubulin alpha-4a chain (TUBA4A), tubulin beta-2A chain (TUBB2A), tubulin alpha-1a (TUBA1A), and beta tubulin (TUBB). Significantly overexpressed in migratory BC cells [32] and identified as a highly expressed gene in primary breast tumors with brain-specific metastasis [118], TUBA4A was found as overexpressed in MCF7 BC cell line in upregulated JTB condition of this experiment. TUBA4A is a member of alpha-tubulin family involved in cellular movement and development [32] by formation of tubulin-based microtentacles as cytoskeletal structures that sustain the metastatic dissemination, in association with EMT pathway [33]. High TUBA1A expression was correlated with mTOR and p38 MAPK pathways, which may control proliferation, growth, and survival of cancer cells; elevated TUBA1A expression was correlated with invasive subtypesand poor overall survival in GC patients [80]. TUBB2A might control the migration of BC cells from a primary tumor to distant metastatic sites by regulation of the adhesion and proliferation of BC cells [34].

**HSPs related to JTB dysregulation promote EMT**. Several heat shock proteins (HSPs) have been reported as EMT inductors associated with increased invasiveness of cancer cells [119]. HSPD1 might repress E-cadherin expression and promotes metastatic characters such as EMT of buccal mucosa squamous cell carcinoma (BMSCC) cells [18]. Also, the upregulated HSP90AA1 promotes the expression of EMT biomarkers in MDA-MB-231 cells [21]. HSPD1 was established as a protein biomarker for metastatic BC related to lymph node metastasis and regional metastasis [34]. The overexpression of HSPA1A chaperone was found to elevate cell motility and to upregulate the EMT biomarkers in colon cancer cells incubated in hyperglycemic condition associated with tumorigenesis [23].

**JTB-related metabolic reprogramming promotes EMT**. EMT process is subjected to the metabolic regulation, while EMT rewires the metabolic program to adapt to cellular changes during EMT [120]. Usually, the EMT-derived BC cells emphasize the overexpression of several enzymes and transporters related to aerobic glycolysis [121]. Thus, the overexpression of enolase 2/neuron-specific enolase (ENO2/NSE), an important glycolytic enzyme, has been reported as an EMT inductor in pancreatic cancer cells, thereby promoting metastasis [122]. Glyceraldehyde-3-phosphate dehydrogenase 1 (GAPDH1), also upregulated in JTB downregulated condition, is known to play an important role in metabolism and gene transcription; it promotes cancer growth and metastasis by affecting EMT through upregulation of SNAIL expression [91]. Playing an important role in tumor metabolism, phosphoglycerate kinase 1 (PGK1), upregulated in JTB downregulated condition in this experiment, under hypoxic conditions, promotes glycolysis and increases stem-cell like abilities and the EMT in oral squamous cell carcinoma (OSCC) cells through the AKT signaling pathway [108]. Peptidyl-prolyl isomerase A/cyclophilin A (PPIA/CYPA) has been reported as overexpressed in BC, promoting cell survival [17], cancer cell growth, malignant transformation, metastasis, drug resistance [38], anti-apoptosis [39], and EMT in NSCLC cells via p38 MAPK [40].

To survive and proliferate in both well oxygenated and hypoxic microenvironments, cancer cells develop three cellular metabolic phenotypes: glycolytic (aerobic glycolysis), oxidative (oxidative phosphorylation), and hybrid, based on both OXPHOS and glycolysis, which are simultaneously activated [123]. Increased glycolysis is commonly exhibited in cancer cells, allowing them to produce energy, known as the Warburg effect [124]. Based on GSEA results, we found that glycolysis-related enzymes, such as ENO2, PPIA, PGK1, and GAPDH1 described above as promoters of EMT, were differentially overexpressed when JTP was dysregulated in MCF7 cells. In contrast, SOD1, that is also an adipogenesis-related enzyme, ENO1, and TPI1 were downregulated. HALLMARK_FATTY_ACID_METABOLISM was found to be significantly upregulated in overexpressed JTB condition in correlation with the overexpression of ENO2, a glycolysis-related enzyme that contributes to the increased fatty acid production [125] and HSP90AA1 that may activate the MTORC1 signaling pathway [107], which is upregulated in multiple cancer types, including BC [126], leading to cell growth and tumor proliferation and playing a significant role in endocrine resistance in BC [127]. The oncogenic signal transduction pathway PI3K-AKT-mTOR regulates fatty acid metabolism [128]. However, the downregulation of the fatty acid synthase (FASN) enzyme has been observed in both dysregulated JTB conditions in this experiment, while an overexpression of FASN has been previously identified in the overexpressed JTB condition in the SDS-PAGE-based proteomics [13]. FASN was reported as highly upregulated in BC cell lines, including the hormone-dependent MCF7 line [129], and in a variety of human cancers in association with invasion and poor prognosis [130]. It is well known that the FASN inhibition could suppress or reduce the proliferation, migration, invasion and induces apoptosis by inhibiting β-catenin and C-Myc in HepG2 hepatoma carcinoma cells [54]. However, it is possible that during the initiation of the EMT process, cancer cells switch from a rapid cell growth and a proliferative state, characterized by high de novo lipid biosynthesis that requires FASN, to a migratory phenotype, in which FA uptake or selective release of FA from membrane lipids leads to the formation of signaling molecules involved in cell migration and invasion [131]. The mitochondrial solute carrier family 25-member 5 (SLC25A5) protein, also known as ANT2, here overexpressed in overexpressed JTB condition, has been reported as positively correlated to the oxidative phosphorylation (OXPHOS). SLC25A5 is known to regulate adipogenesis by modulation of extracellular signal-related kinase (ERK), a member of MAPK signaling pathway [132], that promotes cell proliferation, angiogenesis, cell differentiation, and cell survival [133].

**JTB-related proteins involved in cellular proteostasis may promote EMT**. Enhanced regulation of cellular proteostasis is observed in tumors, suggesting the essential role of proteostasis in tumorigenesis and cancer development [134]. The intracellular pathways that assure the protein quality control are essential for survival of BC cells that are exposed to stressful condition, such as an increased in protein translation or accumulation of unfolded proteins, as well as microenvironmental factors, such as altered pH and glycosylation, oxidative stress (OS), cellular damage, nutrient deprivation, viral infection, and hypoxia [135,136], which lead together to endoplasmic reticulum (ER) stress [135]. Dysregulation in protein synthesis, transport, folding, degradation and secretion in cancer cells lead to overexpression of ER chaperones that facilitate selective degradation of target misfolded proteins through unfolded protein response (UPR) and ubiquitin-proteasome system (UPS) or by autophagy-lysosomal pathways (ALPs) [136].

JTB overexpression was here associated with an alteration in the expression of proteins that have been functionally linked to selective degradation of target misfolded proteins by HALLMARK_UNFOLDED_PROTEIN_RESPONSE (UPR) (RPS14, RPL6, TUBB2A, and PRKCSH) and chaperone-mediated autophagy (CMA) (LAMP2, HSP90AA1, and EEF1A1), promoting proliferation, migration and survival cancer cells in stress condition. RPS14, RPL6, PRKCSH and TUBB2A were detected as overexpressed in overexpressed JTB condition. Ribosomal protein S14 (RPS14) is overexpressed in ER+ BC cells, while its downregulation inhibited cell proliferation and metastasis and induced apoptosis [25]. Protein kinase C substrate 80K-H/Hepatocystin [37]/Glucosidase 2 subunit beta (PRKCSH) contributes to tumorigenesis, being upregulated in various tumors, including BC [37]. PRKCSH is involved in induction of tumor-promoting factors and tumor resistance to ER stress by selective activation of IRE1 branch of UPR [37].

Cancer cells highlight the ability to exploit UPR signaling induced by accumulation of misfolded and unfolded proteins to promote EMT [137]. Many types of cancer cells overexpress CMA as a proteostatic process for activation of protumorigenic and pro-survival pathways by decreasing of the cellular stress level in growing tumors and maintaining of the oncogenic load [138]. However, CMA may develop a favorable impact in cancer progression or could exert an antioncogenic effect by degradation of pro-oncogenic proteins [139]. In overexpressed JTB condition, LAMP2, HSP90AA1, and EEF1A1 proteins are overexpressed, being involved in CMA biological process. As a key receptor protein in CMA pathway, the lysosome-associated membrane protein type 2A (LAMP2A) is present in lysosomal membrane, being usually overexpressed in BC tissues than in corresponding healthy tissues as well as in different cancer cell lines, contributing to their proliferation [35]. It is involved in chaperone-mediated translocation and binding of modified and oxidatively damaged proteins to the lysosomal membrane and formation of a translocation complex that facilitate the internalization of the substrate protein into the lumen of lysosomes for degradation and protein turnover [36]. LAMP2 is also involved in HALLMARK_PROTEIN_SECRETION. Eukaryotic translation elongation factor-1 alpha 1 (EEF1A1) is a protein also involved in cytoskeleton modulation [140] that emphasizes chaperone-like activity and controls cell proliferation and cell death [140]. HSP90AA1 is an essential molecular chaperone overexpressed in tumors that could serve as a cancer biomarker [19]. This protein plays an important role in carcinogenesis, gene expression, regulation of protein folding and assembly of large multiprotein complexes, DNA damage regulation, cell cycle regulation, and activation of oncogenic proteins involved in cancer cell survival, adaptation to stress, growth, proliferation, angiogenesis, signal transduction, metabolic rewiring, motility and invasiveness [20]. Ubiquitin-like protein ISG15/interferon stimulated gene 15 is a member of protein modification pathway that was found to be a novel inhibitor of autophagy, its depletion or downregulation promoting autophagy and cell survival [141]. In downregulated JTB condition, ISG15 was found as downregulated.

Protein misfolding also promotes cancer progression. Heat skock proteins (HSP)60, HSP70 and HSP90, the most identified proteins in proteomic approaches, are involved in protein folding, recognizing target misfolded proteins for degradation [142]. JTB overexpression is also associated with alteration in the expression of proteins that have been functionally linked to protein folding/chaperonin-mediated protein folding (CMPF). Thus, type I chaperonins (HSPD1 and HSPE1), HSPA1A, HSP90AA1, and PRKCSH were found as upregulated in this experiment. The overexpressed heat shock protein family D (HSP60) member 1 (HSPD1) might repress E-cadherin expression, promotes cancer cell invasion, migration [18], and mitochondrial dysfunction, assists protein folding, tracking and degradation, enhances tumor cells survival, while its downregulation induces tumor cell apoptosis in BC cells and cell lines [143]. HSPD1 and heat shock protein family E (HSP10) member 1 (HSPE1) have been cited as overexpressed in basal, HER2 and luminal B, known as the most aggressive subtypes of BC [81]. Heat shock 70 kDa protein 8 (HSPA8) is a chaperone protein that facilitates accurate protein folding; is was found as overexpressed in various cancer cells where it promotes cell growth, proliferation and metastasis, while its depletion suppresses cancer cells growth, induces apoptosis, and cell cycle arrest [96]. Here, HSPA8 as well as HSPB1 were found to be downregulated in JTB downregulated condition. PRKCSH, as mentioned above, ensures secretion of properly folded glycoproteins and degradation of misfolded glycoproteins by endoplasmic reticulum-associated degradation (ERAD) pathway [37].

**JTB-related proteins are involved in ribosome biogenesis linked to EMT**. A strong relation between the EMT program and ribosome biogenesis is known to lead to an increased migration, invasion, and metastasis [144]. Consequently, hyperactivation of ribosome biogenesis and aberrant ribosome homeostasis represent hallmarks of cancer [145]. Ribosome biogenesis is known as a central player in cancer metastasis and therapeutic resistance, cancer cells harboring specific onco-ribosomes that facilitate the oncogenic translation program and promotes metabolic reprogramming [144]. The ribosomal proteins have a well-known role in ribosome integrity and protein synthesis as well as in gene transcription, cell proliferation, apoptosis and differentiation [146]. Several proteins in this experiment are involved in cytoplasmic translation and ribosome assembly/biogenesis. Thus, 40S ribosomal protein S14 (RPS14) and human 60S ribosomal protein L6 (RPL6) were detected as upregulated in overexpressed JTB condition. RPS14, considered as indispensable for ribosomal biogenesis, was highly expressed in ER+ BC tissues compared with ER- tissues, while its downregulation significantly inhibits cell proliferation, cell cycle, and metastasis, inducing apoptosis and activating the interferon signaling pathways [25]. RPL6 was reported as an overexpressed protein in multidrug-resistant gastric cancer cells compared with normal gastric mucosa, this upregulation accelerating growth, enhancing the in vitro colony forming ability of cancer cells and anti-apoptosis, while its downregulation reduced colony forming ability, cell growth, and cell cycle progression [26]. The genetic manipulation of 40S ribosomal protein S5 (RPS5) expression interacts with the cell growth and differentiation [146]. Downregulated in JTB downregulated condition, RPS5 protein is known for the negative regulation of the expression of p53 and for its anti-apoptotic role in cancer cells, conferring resistance to mitogen-activated extracellular signal-regulated kinase (MEK) inhibitor-induced cell death [100]. Translation factor EEF1A1 could act as an oncoprotein that favors cellular transformation through aberrant protein translation associated with cytoskeleton alterations and modulation of signaling pathways [46].

### 2.2. JTB Dysregulation Is Associated with Mitochondrial Organization and Function

Mitochondria are key organelles related to the alterations of the main pathways involved in energy metabolism and biosynthesis that are profoundly dysregulated in cancer cells [44]. There are studies which suggest both stimulative and suppressive impact of mitochondrial function on tumorigenesis, related to tumor stage and microenvironmental conditions [7]. One hypothesis sustains that mitochondrial metabolism has a tumor suppressor function by inhibiting cancer cell proliferation and activating apoptosis, consequently to the overproduction of superoxide radical as a result of the stimulation of mitochondrial metabolism [147]. However, during malignant transformation, the specific cancer cell clones that have stimulated mitochondrial biogenesis are known to have elevated aggressiveness [123]. It is known that JTB affects morphology and membrane potential of mitochondria, the dysregulation in JTB expression or aberrant JTB structure affecting mitochondrial functions in correlation to the metabolic state of the cells and production of superoxide, contributing to malignant transformation of cells [7]. Mitochondrial signaling is involved in cell growth and proliferation, apoptosis and stress response of cells [7], mitochondrial bioenergetics and cell death being tightly connected [147]. Consequently, the oxidative phosphorylation (OXPHOS) and cell death are both the molecularly and functionally integrated major functions of mitochondria [147].

The expression levels of HSPD1, HSP90AA1, HSPA1A, PARK7 and SLC25A5 proteins are associated with mitochondrion organization biological function. HSPD1 is a mitochondrial chaperone overexpressed in cancer cells, which is involved in cell proliferation [148]. Into a HSPD1-centered PPIs network built using the Search Tool for Retrieval of Interacting Genes/Proteins (STRING) for glioblastoma multiforme cells (GMF), HSPD1 was associated with proteins involved in protein folding (such as upregulated HSP90AA1) as well as in metabolic pathways, such as glycolysis and OXPHOS [148]. It is known that chaperonin HSPD1 co-expressed with tricarboxylic acid cycle enzymes, while HSPE1, also upregulated in downregulated JTB condition, co-expressed with proteins involved in OXPHOS [149]. OXPHOS pathway is required for neoplastic transformation of cells and plays a role in tumor metastasis, stemness and drug resistance [150]. OXPHOS is represented here by a member of the mitochondrial carrier subfamily of solute carrier proteins, SLC25A5, that was found to be upregulated in overexpressed JTB condition. Associated with glycolytic metabolism, SLC25A5 inhibits mitochondrial membrane permeability and may act as an anti-apoptotic oncoprotein [45]. This protein sustains cancer cell survival under microenvironmental hypoxia or may lead to instability of mitochondrial genome [44]. The overexpressed PARK7 protein interacts with the anti-apoptotic protein BCL2L1, increasing its mitochondrial localization, with effects in tumorigenesis, cancer cells proliferation, metastasis, recurrence and resistance to chemotherapy [84]. Stress-inducible HSPA1A/HSP70 is abundantly present in mitochondria of tumor cells; its inhibition leads to a loss of mitochondrial membrane potential, promoting mitochondrial dysfunction [150]. HSPA1A and HSP90AA1 have been also identified as overexpressed within our previous experiment based on in-gel proteomic analysis of overexpressed JTB condition in MCF7 BC cell line [13].

### 2.3. JTB-Related Proteins Are Involved in Oxidative Stress (OS)

Cancer cells usually overexpress antioxidant proteins to maintain the redox balance [86]. Elevated reactive oxygen species (ROS) levels in tumor microenvironment (TME) activate tumorigenesis, promote cell proliferation, increase cell survival, induce DNA damage but can also promote antitumor signals and induce tumor apoptosis [86]. PRDX1 downregulation was detected in overexpressed JTB condition. Loss of peroxiredoxin 1 (PRDX1) activates fibroblasts to become invasive cancer-associated fibroblasts (CAFs) by regulation of c-Jun N-terminal kinases/JNK signaling, and promotes cancer development in mammary gland [59]. The peroxidase peroxiredoxin 2 (PRDX2) was identified here as upregulated in downregulated JTB condition. It is found to be overexpressed in many cancers [86], reducing OS, cell damage and apoptosis [88], while its knockdown inhibits cell proliferation, migration, invasion, tumor growth and EMT in lung cancer [151] and colorectal cancer [86]. PARK7 is also a “redox sensor” that protects tumor cells from OS [84].

Downregulated in this experiment, SOD1 does not support oncogene-dependent proliferation, that happened when it is overexpressed and maintains ROS levels below a threshold that support the growth of cancer cells [61]. Annexin A2 (ANXA2), a protein that could a play a role in cellular redox regulation and tumorigenesis [106], was identified as downregulated in downregulated JTB condition. Depletion of ANXA2 resulted in elevation of cellular ROS upon OS, activation of the ROS-induced pro-apoptotic kinases, JNK, p38, and AKT, and increased sensitivity to ROS-mediated cell damage/death, elevated protein oxidation, and decreased tumor growth [106].

### 2.4. JTB-Related Proteins Are Involved in Apoptotic Pathway

Inducing apoptosis is cited as an important factor to control excessive BC cells proliferation [152], apoptosis pathway being frequently dysregulated in cancer development [153]. Silencing of JTB expression has been cited to promote cancer cell motility and emphasized anti-apoptotic effect in hepatocellular carcinoma (HCC) [154].

**JTB-related proteins with anti-apoptotic effect**. Heat shock proteins play a key role in regulation of apoptotic cell death [155]. There are studies that demonstrated that the combined overexpression of HSPE1 and HSPD1, both overexpressed here indownregulated JTB condition, is important for protein folding in mitochondria, emphasizing cellular protective effects by increasing in the anti-apoptotic B-cell lymphoma 2 (BCL-2) expression through post-translational mechanisms [155]. PARK7 protein is overexpressed in downregulated JTB condition in MCF7 BC cell line; like in other various types of cancer, it suppresses apoptosis in tumor cells [84]. When overexpressed, as in this experiment in upregulated JTB condition, RPS14 and RPL6 ribosomal proteins could also protect cancer cells from chemotherapeutic drug-induced apoptosis [26], stimulating tumor cells proliferation, cell cycle, metastasis and anti-apoptosis [25,146], suggesting their protumorigenic function. Also detected here as downregulated in overexpressed JTB condition, reduced IQGAP2 can inhibit apoptosis by modulating the MEK-ERK and p38 signaling in BC [65]. Overexpressed in upregulated JTB condition, SLC25A5, also known as adenine nucleotide translocase 2 (ANT2), acts as an anti-apoptotic oncoprotein [45]. Overexpressed in downregulated JTB condition, proteasome activator complex 1 (PSME1) negatively regulate the apoptotic pathways [73]. Overexpressed in upregulated as well as in downregulated JTB condition, PPIA has been reported to emphasize anti-apoptotic effects [39].

**JTB-related proteins with a pro-apoptotic effect**. RPS5 negatively regulates the expression of p53 and plays an anti-apoptotic role in cancer cells, conferring resistance to mitogen-activated extracellular signal-regulated kinase (MEK) inhibitor-induced cell death [100]. However, in downregulated JTB condition, RSP5 expression was detected as downregulated and, consequently, RSP5 could emphasize an anti-tumor effect as in the case of RPS15-depleted cancer cells that suffer apoptosis under chemotherapy via upregulation of several apoptotic proteins [100]. Inhibition of FASN, a metabolic enzyme also detected in this experiment as downregulated in both dysregulated JTB condition, induced apoptosis by inhibiting β-catenin (CTNNB1)/C-Myc signaling pathway, as well as migration and invasion of HepG2 hepatoma carcinoma cells [54]. CTNNB1 is a component of Wnt signaling pathway that is important in tumorigenesis and plays a key role in most cancers, acting as an oncogene; its knockdown inhibited cell proliferation, migration, and invasion and induced apoptosis in renal cell carcinoma (RCC) [101]. A slowed tumor progression or a significantly increased apoptosis was reported upon MKI67 knockdown/knockout in several cancer cells lines [98]. Tumor protein D52-like 2 (TPD52L2/TPD54) was cited as overexpressed in BC, OC and PCa [63]. Its silencing, also identified in overexpressed JTB condition in this experiment, reduced cell viability, cell colony-forming potency, cell growth, and induces apoptosis and ER stress of oxaliplatin-resistant gastric carcinoma cells [63]. JTB dysregulation in MCF7 cells was associated with downregulation of other proteins that might be associated with pro-apoptotic and anti-tumorigenic effects, such as calcium-calmodulin N-terminal domain 1(CALM1), downregulated here in overexpressed JTB condition, which induces apoptosis in MM cells [60] or ESCC [66], and SOD1, downregulated in both overexpressed and downregulated JTB condition [61]. GAPDH1, upregulated in downregulated JTB condition, is known to have a pro-apoptotic role [73]. Annexin A2 (ANXA2) was identified as downregulated in downregulated JTB condition. Depletion of ANXA2 results in elevation of cellular ROS upon OS, activation of ROS-induced pro-apoptotic kinases, JNK, p38, and AKT and increased sensitivity to ROS-mediated cell damage/death [106].

### 2.5. JTB-Related Proteins Are Involved in Interferon Alpha and Gamma Signaling Pathways

Interferons play an essential role in the immune landscape of BC [156]. Even if IFN-α signaling pathway contributes to apoptosis and cellular senescence, in contrast, it also could play a role in increased migration and drug resistance, depending on the interferon-stimulated transcribed genes [157]. Interferon gamma also plays a dual tumor-suppressor and protumorigenic roles in cancer [158]. In inflammatory breast cancer (IBC) cells, the increased levels of IFN-α has been reported as protumorigenic factors involved in IBC progression [157]. IFN-γ plays a key role in the regulation of antitumor immunity, but it also develops a protumorigenic role by proliferative and anti-apoptotic signals that lead to immune-escape of cancer cells [156] and stimulation of tumor progression and metastasis [159]. It is demonstrated that the IFN-α signaling activation in the tumor cells alters the phenotype of immune and stromal cells within the tumor-associated stroma [157], enhancing cancer cells motility and invasion and promoting BC metastasis [160]. JTB dysregulation is here associated with alteration in the expression of proteins that have been linked toHALLMARK_INTERFERON_ALPHA_RESPONSE and HALLMARK_INTERFERON_GAMMA_RESPONSE that are significantly upregulated in downregulated JTB condition, correlated with the expression of interferon-induced transmembrane protein 2 (IFITM2), proteasome activator complex subunit 1 (PSME1) and interferon-stimulated protein 15/ubiquitin-like protein ISG15.

IFITM2 was found as upregulated in overexpressed JTB condition and downregulated in JTB downregulated condition in MCF7 BC cell line. IFITM2 was reported to sustain tumor progression and lymphatic metastasis by inducing cytokines release, while migration and invasion were inhibited by the IFITM2 downregulation in renal clear cell renal carcinoma (ccRCC) [27]. However, knocking out IFITM2 could enhance the activation of the endogenous IFN-α pathway that may alter the immune and stromal cells in the TME enhancing the invasive abilities of cancer cells [99]. In downregulated JTB condition, PSME1 was found to be upregulated. PSME1 may play different roles in various types of cancer [71]. Thus, PSME1 has been identified as tumor-associated protein/putative tumor biomarker/upregulated in human esophageal squamous cell carcinoma (hESCC) [69], primary and metastatic human prostate cancer (PCa) [70], skin cutaneous melanoma (SKCM) [71], and multiple myeloma (MM) [72], while it was reported as downregulated in hepatitis B virus-infected well-differentiated hepatocellular carcinoma (HCC) [74]. In SKCM, PSME1 was positively correlated with apoptotic process, cell adhesion, cell cycle, metastasis, NF-κB and Wnt signaling pathways [71]. When upregulated, it is involved in melanoma cell growth and proliferation [72]. ISG15is usually overexpressed in BC cells, but it was downregulated in this experiment. The aberrant activation of the ISG15 leads to a higher motility of tumor cells by disrupting cytoskeletal architecture and stabilizing proteins that contributes to cell motility, invasion and metastasis [161]. However, ISG15 was reported as an endogenous tumor suppressor but, when dysregulated in cancer cells, may be subverted to promote tumorigenesis [162].

## 3. Discussion

Our previously published results based on in-gel proteomics analysis of transfected MCF7 breast cancer cell line emphasized that the HALLMARK_EPITHELIAL-MESENCHYMAL_TRANSITION (EMT) was the main upregulated pathway in both overexpressed [13] as well as in downregulated JTB condition [14]. In JTB overexpressed condition, we previously identified FLNA, COL11A, and COL3A1 as upregulated proteins directly involved in EMT pathway and included in GSEA algorithm. Taking account that in-solution digestion-based proteomics experiments are complementary to the initial gel-based ones, in this experiment based on in-solution proteomics we identified and analyzed as overexpressed several complementary proteins involved in promotion of the EMT program. Thus, some proteins involved in cytoskeleton organization and modulation could play a pivotal role in EMT. We focused on the participation of actin filaments and microtubules-related proteins in promoting EMT and their influence on cancer metastasis, such as TMSB10, TPM3, IQGAP2, ACTC1, ACTG1, ACTN4, TUBA4A, TUBB, TUBA1A, TUBB2A, POTEKP/ACTBL3, and EEF1A1. It is well-known that EMT is subjected to the metabolic regulation, while EMT rewires the metabolic program. Thus, we identified the overexpression of many glycolysis-related enzymes reported to sustain the EMT process or to increase the stem-cell like abilities in tumoral cells, such as ENO2, GAPDH, PGK1, and PPIA. Some enzymes involved in HALLMARK_FATTY_ACID_METABOLISM, also upregulated in overexpressed JTB condition, were found as upregulated, such as ENO2, above mentioned as a glycolytic enzyme that also contributes to the increased fatty acid production, and HSP90AA1 that also may activate MTORC1 signaling pathway, usually overexpressed in BC cells. Interestingly, FASN enzyme that has been found as upregulated in in-gel proteomics analysis, was identified as downregulated by using in-solution digestion. Usually overexpressed in cancer cells, FASN downregulation could be associated with the switch of MCF7 cancer cells between a proliferating state to a migratory behavior that is based on an exogenous uptake of fatty acids and FAs release from cell membranes rather than an intracellular de novo synthesis of FAs. We can also conclude that JTB-related proteins are involved in cellular proteostasis and ribosomal biogenesis, both processes sustaining the EMT. JTB dysregulation was also associated with HALLMARK_MITOTIC_SPINDLE upregulation, mitochondrial organization and function, oxidative stress, apoptotic pathway and interferon alpha and gamma response. Consistent and complementary to our previous results emerged by in-gel based proteomics of transfected MCF7 cells, JTB-related proteins that are overexpressed in this experiment suggest the development of a more aggressive phenotype and behavior for this luminal type A non-invasive/poor-invasive human BC cell line that does not usually migrate or invade compared with the highly metastatic MDA-MB-231 cells. However, in both JTB dysregulated conditions, several downregulated JTB-interacting proteins predominantly sustained antitumor activities, attenuating the aggressive phenotypical and behavioral traits promoted by the overexpressed JTB-related partners. It is necessary to put together all data obtained by using in-gel (SDS-PAGE and 2D-PAGE) and in-solution proteomics applied to transfected MCF7 BC cells and other analyzed cell lines to conclude if JTB could be used as a new biomarker in breast cancer.

## 4. Materials and Methods

### 4.1. Cell Culture

MCF7 cells were ordered from American Type Culture Collection (HTB-22 ATCC) and RPMI media supplemented with 10% FBS, 1% Penicillin Streptomycin, 0.2% Amphotericin and 0.2% Gentamicin (growth media) was used for their growth. The cells were incubated at 37 °C in 5% CO_2_. The media was replaced every 48 h and they were allowed to reach ~70% confluency.

### 4.2. Plasmids for Upregulation

**Figure 1:**
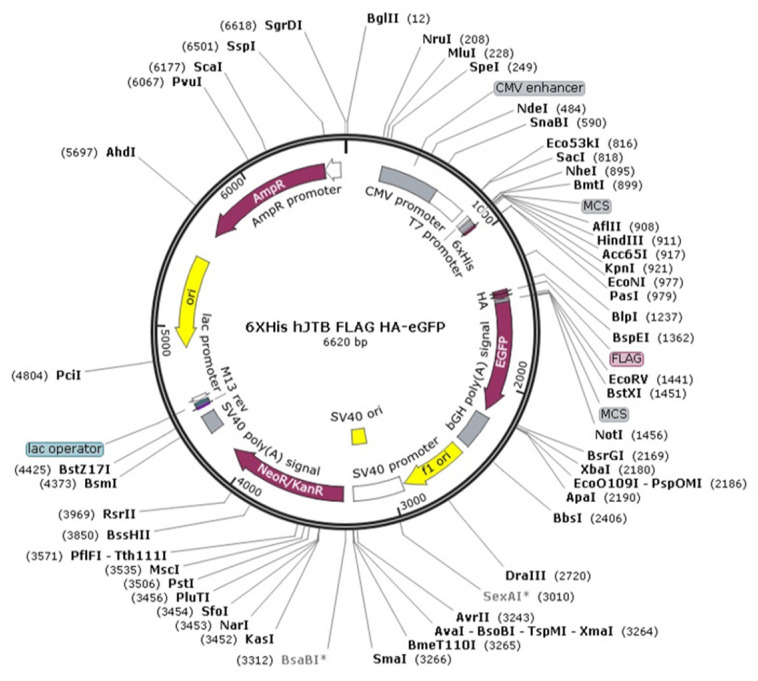
Plasmid for upregulation from Creative Biogene with customized 6X His tag at the N-terminus, HA, FLAG and eGFP tag at the C-terminus.

Two plasmids were custom made by Genscript, Piscataway, NJ, USA^®^. One plasmid was an empty vector with an eGFP tag to serve as control and the other plasmid with hJTB gene containing the full coding region of cDNA, ggtaccGCCACCATGCATCATCATCATCATCATCTTGCGGGTGCCGGGAGGCCTGGCCTCCCCCAGGGCCGCCACCTCTGCTGGTTGCTCTGTGCTTTCACCTTAAAGCTCTGCCAAGCAGAGGCTCCCGTGCAGGAAGAGAAGCTGTCAGCAAGCACCTCAAATTTGCCATGCTGGCTGGTGGAAGAGTTTGTGGTAGCAGAAGAGTGCTCTCCATGCTCTAATTTCCGGGCTAAAACTACCCCTGAGTGTGGTCCCACAGGATATGTAGAGAAAATCACATGCAGCTCATCTAAGAGAAATGAGTTCAAAAGCTGCCGCTCAGCTTTGATGGAACAACGCTTATTTTGGAAGTTCGAAGGGGCTGTCGTGTGTGTGGCCCTGATCTTCGCTTGTCTTGTCATCATTCGTCAGCGACAATTGGACAGAAAGGCTCTGGAAAAGGTCCGGAAGCAAATCGAGTCCATAGACTACAAAGACGATGACGACAAGTACCCATACGATGTTCCAGATTACGCTgatatc corresponding to 146 amino acids of the protein was made. The hJTB cDNA was inserted into a CMV promoter based plasmid in the sense orientation to get the JTB overexpression. This plasmid was further customized with His, HA, FLAG and an eGFP tag to enable confirmation of transfection efficiency (Figure 1).

### 4.3. Plasmids for Downregulation

**Figure 2:**
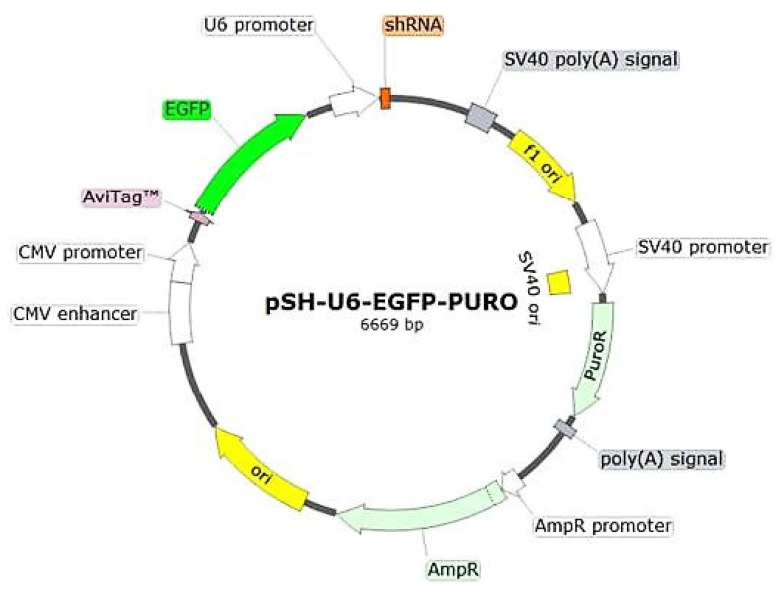
Plasmid for downregulation from Creative biogene.

Four shRNA plasmids were custom made by Creative Biogene, Shirley, NY, USA. One plasmid containing the scramble shRNA sequence GCTTCGCGCCGTAGTCTTA to be used as control. Three shRNA plasmids targeting the hJTB sequence GCTTTGATGGAACAACGCTTA, GCAAATCGAGTCCATATAGCT, GTGCAGGAAGAGAAGCTGTCA respectively were customized into a psh-u6-egfp-Puro vector, containing an U6 promoter, a His tag and an egfp tag with a puromicin resistance gene. Three plasmids targeting different hJTB sequences were made in order to get successful downregulation of JTB in case one of them did not work efficiently (Figure 2).

### 4.4. Transfection into MCF7 Cells

DNA/Plasmid (10 µg/µL) and Lipofectamine™ 3000/DNA complexes were prepared in Opti-MEM reduced serum media (Invitrogen, Waltham, MA, USA) for each condition and added directly to the cells in culture medium once they reached 70% confluency. Stable transfection was performed for overexpressed JTB condition, where 2 mg/mL of Neomicin was added to the growth media after 48 h of transfection and the media containing the antibiotic was replaced every two days. The cells that survived were allowed to reach 80% confluency. The cells were observed under the confocal microscope to visualize green fluorescence from the eGFP protein, which confirmed transfection efficiency. Transient transfection was performed on downregulated JTB conditions, where the cells were collected after three days of transfection, after visualizing the eGFP fluorescence under the microscope.

### 4.5. Western Blot Analysis

Lysis buffer containing 20 mM Tris HCl, 0.2 mM EDTA, 150 mM NaCl, protease & phosphatase inhibitors and 1.1% Triton-X were used to collected cell lysates from each condition. The lysates were incubated on ice for 30 min and centrifuged at 4 °C for 20 min at 14,000× *g* rpm. Bio-Rad protein assay dye with BSA standards was used to determine the protein concentration of the supernatants. 20 µg of proteins were run in a 14% SDS-PAGE gel and transferred to a nitrocellulose membrane. The blots were incubated with blocking buffer containing 5% milk and 0.1% tween-20 overnight at 4 °C with shaking. Primary antibody (JTB Polyclonal Antibody—PA5-52307, Invitrogen, Waltham, MA, USA) diluted to 1:1000 was added and incubated at 4 °C for 1 h with constant shaking. Secondary antibody (mouse anti-rabbit IgG-HRP sc-2357, Santa Cruz Biotechnlogy, Inc., Dallas, TX, USA) diluted to 1:2000 ratio was added and incubated for 1 h at room temperature with constant shaking. After each incubation, the blots were washed thrice with TBS-T (1 X TBS buffer, containing 0.05% tween-20) for 10 min each with constant shaking. Finally, the enhanced chemi-luminescence substrate (Pierce™ ECL Western Blotting Substrate—32106, ThermoFisher, Waltham, MA, USA) was added to the blot and the blot was analyzed using a CCD Imager. For normalization, the blots were treated with Mouse GAPDH monoclonal antibody (51,332, Cell-Signaling Technology, Danvers, MA, USA) and incubated for 1 h, followed by 1 h incubation of goat anti-mouse IgG-HRP (sc-2005, Santa Cruz Biotechnology) and the addition of ECL substrate. Detection and comparison of the intensity of the bands were done using ImageJ software.

### 4.6. In-Solution Digestion

200 µg of proteins for four samples in three biological replicates: control, upregulated JTB, shRNA control and downregulated JTB were dried down in a Speedvac and resolubilized in 20 µL of 6 M urea, 100 mM Tris Buffer. The samples were sonicated for 30 min. 1 µL of the reducing agent containing 200 mM DTT and 100 Mm Tris was added and the sample was gently vortexed and allowed to incubate at room temperature (RT) for 1 h. 4 µL of the alkylating agent containing 200 mM IAA and 100 mM Tris was added to the sample, gently vortexed and incubated at RT for one hour in the dark. 4 µL of the reducing agent was added again and incubated at RT for one hour after gentle vortex. 155 µL of water was added to reduce urea concentration and 20 µL of trypsin solution (containing 4 µg trypsin). With a 1:20 ratio of trypsin to protein. The sample was then incubated overnight at 37 °C. The reaction was stopped by adjusting the pH to <6 by addition a few drops of concentrated acetic acid. They were then completely dried in the Speedvac. The samples were then solubilized in 100 µL of 0.1% FA and ziptipped using 1 mg ziptip (Glygen, Baltimore, MD, USA) and dried down. Finally, resolubilization was done in 2% ACN and 0.1% FA for LC-MS/MS analysis. All samples were run in triplicates.

### 4.7. MS Analysis

Nanoacquity liquid chromatography (LC) and MS (LC-MS/MS) was used to analyze the peptide mixture in NanoAcquity UPLC (Waters, Milford, MA, USA) coupled to a QTOF Xevo G2 MS (Waters) according to the procedures mentioned in [163]. 100 µM × 10 mm NanoAcquity BEH130 C18 1.7 µm UPLC column (Waters) was used to load the peptides and eluted over 240-min gradients at a flow rate of 400 nL/min as follows: 1% organic solvent B (ACN containing 0.1% FA) over 1–20 min, 8% B (20–150 min), 20–45% B (150–220 min), 90% B (230–240 min). HPLC water in 0.1% FA was used as the aqueous solvent A. The column was connected to a Picotip Emitter Silicatipnano-electrospray needle (New Objective, Woburn, MA, USA) [163]. MS data acquisition involved survey 0.2 s, 0.5 s for 240-min gradient. MS scans (*m*/*z* range (350–1800 Da) and automatic DDA analysis of the top six ions with the highest intensity, with the charge of 2+, 3+,4+, 5+ and 6+. The MS/MS recorded over *m*/*z* of 50–2000 was triggered when the MS signal intensity over 350 counts/s. The six most intense peaks were selected for CID in the survey MS scans, and ten most intense peaks for the 240-min gradient and fragmented until the MS/MS ion counts reached 6000 or for upto 0.45 s for the 240-min gradient. The procedure used was previously described in [163,164]. Each sample were run three times, giving three technical replicates. 1 pmol Glu1-Fibrinopeptide B (Glufib) standard peptide calibration was performed for both precursor and product ions containing the sequence EGVNDNEEGFFSAR and monoisotopic doubly charged peak with the *m*/*z* of 785.84 [163].

### 4.8. Data Processing and Protein Identification

**Figure 3:**
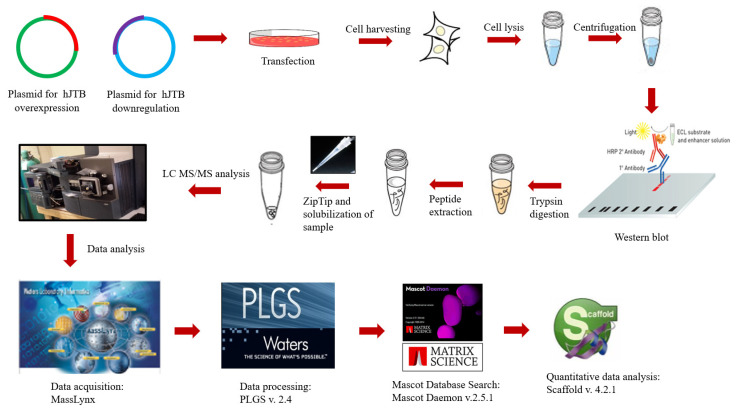
Workflow for cellular proteomics for in-solution digestion.

The raw data obtained from Masslynx software were processed in ProteinLynx Global Server (PLGS, version 2.4, Waters Corporation, Milford, MA) software, as described previously [165,166]. The following parameters were used: background subtraction of five adaptive polynomial order with a 30% threshold, three-channel window with two smoothings in Savitzky-Golay mode and centroid calculation of top 80% of peaks based on four channels with minimum peak width at half height. The resulting peak list (pkl) files were searched against the human database for protein identification in the in-house Mascot server (www.matrixscience.com (accessed on 12 October 2021), Matrix Science, London, UK, version 2.5.1) using the following parameters: human databases from NCBI, 0.5% mass error of Da, 0.8 product ion error of Da, enzyme used: trypsin with three missed cleavages and carbamidomethyl cysteine, methionine oxidized and propionamide cysteine as variable modifications. A list of proteins was obtained for each sample that corresponds each gel band. These data files were then uploaded into Scaffold version 4.2.1 software (Proteome software, Inc., Portland, OR, USA) for quantitative analysis [165] (Figure 3).

### 4.9. Data Sharing

Raw data from Masslynx, HTML files from Mascot and Scaffold files will be provided upon request, according to Clarkson University Material Transfer Agreement.

### 4.10. Statistical Analysis

Data are presented as mean ± S.E.M. Statistical comparisons were made using the three means using paired student’s t-test where appropriate *p* values < 0.05 was considered statistically significant.

### 4.11. Gene Set Enrichment Analysis

The GSEA analysis (https://www.gsea-msigdb.org/gsea/index.jsp (accessed on 27 October 2022)) was conducted to study the hJTB related pathways and biological processes associated with the protein dysregulations in control and upregulated JTB conditions as well as control vs. downregulated JTB conditions in MCF7 cells. The corresponding genes for the dysregulated proteins and their fold change was used for the Hallmark enrichment (h.all.v.7.4.symbols.gmt) with 1000 number of permutations and with 500 maximum size to exclude larger sets and 3 minimum size to exclude smaller sets. The gene set summary obtained from the analysis indicates whether the biological pathways identified are upregulated or downregulated.

## Figures and Tables

**Table 1:** Significant up and downregulated pathways in JTB upregulated condition in MCF7 BC cells, according to GSEA analysis with FDR < 25%.

	Pathways	NES	FDR q-Val
Upregulated	MITOTIC SPINDLE	1.23	1
	EPITHELIAL MESENCHYMAL TRANSITION	1.21	1
	FATTY ACID METABOLISM	1.21	0.979
	UV RESPONSE UP	1.16	0.964
	IL2 STAT5 SIGNALING	1.15	0.82
	APOPTOSIS	1.04	1
Downregulated	OXIDATIVE PHOSPHORYLATION	−1.25	0.138
	APICAL JUNCTION	−1.25	0.127

NES-normalized enrichment score; FDR q-val-false discovery rate q-value.

**Table 2:** Significant up and downregulated pathways in JTB downregulated condition in MCF7 BC cells, according to GSEA analysis with FDR < 25%.

	Pathways	NES	FDR q-Val
Upregulated	COMPLEMENT	1.23	1
	INTERFERON GAMMA RESPONSE	1.07	1
	UNFOLDED PROTEIN RESPONSE (UPR)	1	1
Downregulated	CHOLESTEROL HOMEOSTASIS	−1.56	0.167
	GLYCOLYSIS	−1.45	0.239
	E2F TARGETS	−1.44	0.187
	APICAL JUNCTION	−1.32	0.297
	HYPOXIA	−1.18	0.465
	MYC TARGETS V2	−1.09	0.534

NES-normalized enrichment score; FDR q-val-false discovery rate q-value.

**Table 3:** Deregulated proteins, tumorigenic roles, and biological processes expressed in response to JTB dysregulation.

Gene Symbol	Gene Description	Expression in Malignancies and Putative Neoplastic Effects		HALLMARK_PATHWAYS	GOBP
Overexpressed JTB Condition					
Upregulated proteins and pathways					
HSPD1	Heat shock protein family D (HSP60) member 1	cancer cell survival, regulation of cell death, proliferation [17], represses E-cadherin, promotes cell invasion, migration, poor prognosis [18]	PT	MYC_TARGETS_V1	BIOLOGICAL_ADHESION; PROTEIN_FOLDING; PROTEIN_REFOLDING; PROTEIN_MATURATION; PROTEIN_CONTAINING_COMPLEX_ORGANIZATION; PROTEIN_STABILIZATION; PROTEIN_INTRACELLULAR_TRANSPORT; TRANSMEMBRANE_TRANSPORT; PROTEIN_TRANSMEMBRANE_TRANSPORT_INTO_INTRACELLULAR_ORGANELLE; MITOCHONDRION_ORGANIZATION; DNA_RECOMBINATION; IMMUNE_RESPONSE_TO_TUMOR_CELL; PROGRAMMED_CELL_DEATH
				MTORC1_SIGNALING	
				EMT [18]	
HSP90AA1	Heat shock protein 90 alpha family class A member 1	overexpressed in tumors [19], carcinogenesis, activation of oncogenic proteins involved in cancer cell survival, adaptation to stress, growth, proliferation, angiogenesis, signal transduction, metabolic rewiring, motility and invasiveness [20]	PT	FAM	CELL_MORPHOGENESIS; CELL_PROJECTION_ORGANIZATION; CMA & PROTEIN_CATABOLIC_PROCESS; PROTEIN_FOLDING; PROTEIN_STABILIZATION;PROTEIN_CONTAINING_COMPLEX_ORGANIZATION; MITOCHONDRION_ORGANIZATION; TELOMERE_ORGANIZATION; PROGRAMMED_CELL_DEATH
				EMT [21]	
HSPA1A	Heat shock 70 kDa protein 1A variant	potential biomarker for BC, overexpressed in BC, promotes progression, inhibits apoptosis, extracellularly-activates proinflammatory immunity [22]	PT	COMPLEMENT	REGULATION_OF_CELL_DIFFERENTIATION; MITOTIC_SPINDLE_ORGANIZATION; MITOTIC_CELL_CYCLE_PROCESS; CYTOSKELETON_ORGANIZATION; PROTEIN_FOLDING/CMPF; PROTEIN_REFOLDING; PROTEIN_STABILIZATION; LYSOSOMAL_TRANSPORT; PROTEIN_CATABOLIC_PROCESS; RNA-CATABOLIC_PROCESS; MITOCHONDRION_ORGANIZATION; REGULATION_OF_DNA_TEMPLATED_TRANSCRIPTION_IN_RESPONSE_TO_STRESS PROGRAMMED_CELL_DEATH
				EMT [23]	
RAN	RAS-related nuclear protein/GTP-binding nuclear protein RAN	BC progression, associated with histological grade of tumor, nerve invasion and metastasis, vascular metastasis and Ki-67 [24]	PT	MYC_TARGETS_V1	INTRACELLULAR_PROTEIN_TRANSPORT; MITOTIC_SPINDLE; MITOTIC_CELL_CYCLE_PROCESS; CHROMOSOME_SEGREGATION; RIBOSOME_BIOGENESIS
				E2F_TARGETS	
RPS14	40S ribosomal protein S14	overexpressed in ER+ BC, enhances cell proliferation, cell cycle, metastasis, anti-apoptotic affect, stimulates interferon signaling pathways [25]	PT	UPR	CYTOPLASMIC_TRANSLATION; RIBOSOME_BIOGENESIS/RIBOSOME_ASSEMBLY
RPL6	Human 60s ribosomal protein L6	up-regulated in multidrug-resistant gastric cancer cells, overexpression is anti-apoptotic, accelerates cell growth and colony forming ability [26]	PT	MYC_TARGETS_V1	GOBP_CYTOPLASMIC_TRANSLATION; RIBOSOME_BIOGENESIS/RIBOSOME_ASSEMBLY; Peptide chain elongation (genecards.org)
IFITM2	Interferon- induced transmembrane protein 2	tumor progression and lymphatic metastasis in ccRCC [27]	PT	IFN-α_RESPONSE	DEFENSE_RESPONSE; RESPONSE_TO_INTERFERON_ALPHA; RESPONSE_TO_INTERFERON_BETA; RESPONSE_TO_INTERFERON_GAMMA
				IFN-γ_RESPONSE	
TPM3	Tropomyosin alpha-3 chain	overexpressed in BC, promotes cancer cell migration [28], proliferation, invasion, EMT [29]	PT	EMT [30]	CYTOSKELETON_ORGANIZATION
TUBA4A	Tubulin alpha-4a chain	oncogenic role, drug resistance [31], cell movement and development [32], microtentacles formation and metastatic dissemination [33]	PT	MITOTIC_SPINDLE	MITOTIC_CELL_CYCLE; CYTOSKELETON_ORGANIZATION
				MTORC1_SIGNALING	
				UV_RESPONSE_UP	
TUBB2A	Tubulin beta-2A chain	overexpressed in invasive BC cell lines, predictive biomarker for distant metastasis in BC, cell proliferation, movement, adhesion [34]	PT	UPR	MITOTIC_CELL_CYCLE; CYTOSKELETON_ORGANIZATION; CELL_MIGRATION
				TNFA_SIGNALING_VIA_NFKB	
LAMP2	Lysosome-associated membrane protein type 2	overexpressed in BC tissue and BC cell lines, promotes proliferation [35], protein degradation and turnover [36]; co-overexpressed with HSPA8, promotes cancer cell survival during OS [35]	PT	COMPLEMENT	LYSOSOMAL_TRANSPORT; CMA & PROTEIN_CATABOLIC_PROCESS
				PROTEIN_SECRETION	
				COAGULATION	
PRKCSH	Protein kinase C substrate 80K-H/Hepatocystin [37]/Glucosidase 2 subunit beta	promotes tumorigenesis, overexpressed in tumors, correlated with the progression of lymph node metastasis in BC; induction of tumor-promoting factors and tumor resistance to ER stress [37]	PT	UPR, ERAD pathway [37]	CARBOHYDRATE_DERIVATIVE_METABOLIC_PROCESS; Metabolism of proteins; protein N-glycosylation processing phase (genecards.org);
PPIA/CYPA	Peptidyl-prolyl isomerase A/cyclophilin A	overexpressed in BC, cell survival [17], growth, malignant transformation, metastasis, drug resistance [38], anti-apoptosis [39]; promotes EMT in NSCLC cells [40]	PT	GLYCOLYSIS	CELL_ADHESION; CELL_MIGRATION; PROTEIN_FOLDING; PROTEIN_MODIFICATION_BY_SMALL_PROTEIN_CONJUGATION_OR_REMOVAL; CELL_DEATH_IN_RESPONSE_TO_OXIDATIVE_STRESS
				MYC_TARGETS_V1	
				EMT [40]	
ENO2	Neuron–specific enolase	promotes cell proliferation, glycolysis [41]; overexpressed in lung cancer [42] and glycolytic subtype of TNBC [43]	PT	GLYCOLYSIS	CARBOHYDRATE_METABOLIC_PROCESS
				HYPOXIA	
				APOPTOSIS	
				EMT	
				FAM	
				UV_RESPONSE_UP	
SLC25A5/AAC2/ANT2	Solute carrier family 25 member 5/mitochondrial ADP/ATP carrier-2/adenine nucleotide translocase 2	overexpressed in cancer cells, including BC, induces cellsurvival in hypoxic condition, depletion inhibits tumor cell growth and proliferation, stimulates apoptosis, and facilitates chemotherapy-induced apoptosis [44,45]	PT	OXPHOS	MITOCHONDRION_ORGANIZATION; AUTOPHAGY_OF_MITOCHONDRION; CHROMOSOME_SEGREGATION; NUCLEOTIDE_TRANSMEMBRANE_TRANSPORT; PROGRAMMED_CELL_DEATH
EEF1A1	Eukaryotic translation elongation factor-1 alpha-1	overexpressed in tumors, including BC, controls cell proliferation and cell death [46], promotes heat shock response, protecting cancer cells from proteotoxic stress, sustains cancer cell survival [47], oncogenesis, pro-apoptotic/anti-apoptotic activity	PT	-	CMA & PROTEIN_CATABOLIC_PROCESS; TRANSLATIONAL_ELONGATION; Cytoskeleton modulation
CAND1	Cullin-associated and neddylation dissociated protein 1	overexpressed in PCa, promotes cell viability, proliferation, anti-apoptotic role [48]; mediates invasion and metastasis in ER+ BC through activation of estrogen and androgen signaling pathways [49]	PT	-	REGULATION_OF_DNA_TEMPLATED_TRANSCRIPTION_INITIATION; Centriole duplication control [48]
CREBZF	CREB/ATF bZIP transcription factor	putative tumor-suppressive activity, participates in modulation of p53 [50], reduces MCF7 cell proliferation, migration, and invasion, its knockdown facilitating BC development [51]	AT	-	GOBP_CHROMATIN_ORGANIZATION
Downregulated proteins and pathways					
FASN	Fatty acid synthase	overexpressed in cancer cells, enhances cancer malignant progression [52], tumor cell migration, metastasis [53]; inhibition reduces cell proliferation, suppresses migration and invasion and induces apoptosis [54]	AT	FAM	LIPID_BIOSYNTHETIC_PROCESS; FATTY_ACID_BIOSYNTHETIC_PROCESS; FATTY_ACID_METABOLIC_PROCESS
				CHOLESTEROL_HOMEOSTASIS	
				ESTROGEN_RESPONSE_EARLY	
TPI1	Triosephosphate isomerase	upregulated in multiple cancers, promotes tumor development and progression of BC in tissue and cell lines, promotes glycolysis, proliferation, metastasis, activates PI3K/Akt/mTOR, regulates EMT [55]	AT	GLYCOLYSIS	CARBOHYDRATE_METABOLIC_PROCESS
				MTORC1_SIGNALING	
				HYPOXIA	
PRDX1	Peroxiredoxin-1	regulates cell growth, differentiation, apoptosis, overexpressed in BC tissues and cell lines, controversial role, it could act as tumor suppressor or as a suppressor of tumor cell death [56]; knockout inhibits in vivo growth of mammary tumors derived from MCF7 cells and reduces survival of MCF7 cells under stress condition [57]; tumor suppressor in BC, its deletion promotes tumor growth in mice [58]; loss of PRDX1 results in development of cancer-associated fibroblasts (CAFs) in BC [59]	AT [57]	PEROXIZOME	CELL_REDOX_HOMEOSTASIS
				ROS	
PDIA4	Protein disulfide isomerase A4	upregulated in BC, inhibition promotes reduction of OC cells growth and proliferation, induces apoptosis in MM cells [60]	AT	-	PROTEIN_FOLDING
SOD1	Superoxide dismutase 1 (Cu-Zn)	downregulation promotes apoptosis and oncogene-induced senescence [61]	AT	ROS	CYTOSKELETON_ORGANIZATION; CELL_DEATH_IN_RESPONSE_TO_OS; LIPID_METABOLIC_PROCESS; INFLAMMATORY_RESPONSE; PROGRAMMED_CELL_DEATH
				GLYCOLYSIS	
				PEROXIZOME	
				PROTEIN_SECRETION	
ENO1	Alpha-enolase	overexpressed in BC, involved in cell growth, hypoxia tolerance, autoimmune activities, glycolysis pathway [62]	AT	GLYCOLYSIS	CARBOHYDRATE_METABOLIC_PROCESS
				HYPOXIA	
				MTORC1_SIGNALING	
TPD52L2	Tumor protein D52-like 2	overexpressed in BC, OC and PCa; its knockdown suppressed cell colony-forming potency, cell growth, and induces apoptosis and ER stressof oxaliplatin-resistant gastric carcinoma cells [63]	AT	ANDROGEN_RESPONSE	CARBOHYDRATE_METABOLIC_PROCESS
ACTN4	Actinin alpha 4	BC tumorigenesis, cell movement, proliferation, metastasis; depletion results in reduced proliferation, migration and metastasis, decreases estrogen-mediated cancer cell proliferation in MCF7 [64]	AT	APICAL_JUNCTION	CELL_MORPHOGENESIS; CYTOSKELETON_ORGANIZATION; TRANSMEMBRANE_TRANSPORT
				MITOTICSPINDLE	
IQGAP2	RAS GTP-ase-activating-like protein	tumor suppressor in most cancers, downregulation promotes proliferation and EMT, inhibits apoptosis, stimulates metastatic abilities of BC cells and lymphovascular invasion [65]	PT	ANDROGEN_RESPONSE	CYTOSKELETON_ORGANIZATION; ACTIN_CYTOSKELETON_REORGANIZATION; ACTIN_FILAMENT_BASED_PROCESS
CALM1	Calcium-calmodulin N-terminal domain	knockdown inhibits proliferation, invasion, migration, induces cell cycle arrest and increases apoptosis in ESCC [66]	AT	COMPLEMENT	CYTOSOLIC_CALCIUM_ION_TRANSPORT; TRANSMEMBRANE_TRANSPORT; CELL_CYCLE_PROCESS; MITOTIC_CELL_CYCLE; CYTOKINESIS
AHSG	Fetuin-A/Alpha2- Heremans Schmid (HS) glycoprotein	synthetized, modified and secreted by tumor cells, downregulated, reduces growth, motility, adhesion and attachment of tumor cells [67]	AT	-	INFLAMMATORY_RESPONSE; VESICLE_MEDIATED_TRANSPORT; cell attachment [67]
EEF1A1	Eukaryotic translation elongation factor-1 alpha-1	overexpressed in tumors, including BC, controls cell proliferation and cell death [46], promotes heat shock response, protecting cancer cells from proteotoxic stress, sustains cancer cell survival [47], oncogenesis, pro-apoptotic/anti-apoptotic activity	AT	-	CMA &PROTEIN_CATABOLIC_PROCESS; TRANSLATIONAL_ELONGATION
PCBP1/ hn-RNP-E1 (HNRNP E1)	polyC-RNA-binding protein 1/heterogeneous nuclear riboproteinE1	tumor suppressor, downregulated in human cancers promotes proliferation, migration and invasion of LUAD [68]	PT	-	REGULATION_OF_DNA-TEMPLATED TRANSCRIPTION
**Downregulated JTB condition**					
Upregulated proteins and pathways					
PSME1/ PA28α	Proteasome activator complex subunit 1 isoform 4	tumor-associated protein/putative tumor biomarker/upregulated in hESCC [69], PC [70], SKCM [71], MM, when promotes cell growth and proliferation [72], anti-apoptotic [73]; downregulated in HCC [74]	PT	IFN-α_RESPONSE	PROTEIN_CATABOLIC_PROCESS; MITOTIC_CELL_CYCLE
				IFN-γ_RESPONSE	
ENO2	Neurone –specific enolase	promotes cell proliferation, glycolysis [41]; overexpressed in lung cancer [42] and glycolytic subtype of TNBC [43]	PT	GLYCOLYSIS	CARBOHYDRATE_METABOLIC_PROCESS
				HYPOXIA	
				APOPTOSIS	
				FAM	
				EMT	
				UV_RESPONSE_UP	
PGK1	Phosphoglycerate kinase 1	overexpression associated with poor prognosis in BC, progression, metastases, potential survival biomarker and invasion promoter, regulates HIF-1α-mediated EMT [75]	PT	GLYCOLYSIS	CARBOHYDRATE_METABOLIC_PROCESS
				MTORC1-SIGNALING	
				HYPOXIA	
POTEKP/ACTBL3	POTE ankyrin domain family member K/beta-actin-like protein 3	involved in HCC [76], upregulated in HGSC [77]; lung cancer exosome-specific protein [78]	PT	-	CYTOSKELETON_ORGANIZATION
TUBA1A	Tubulin alpha-1a	upregulated in BC tissue [79], involved in cell division and cell movement; overexpression was correlated with poor overall survival and a more aggressive phenotype in GC [80]	PT	-	MICROTUBULE_BASED_PROCESS; CELL_DIVISION; CELL_JUNCTION_ORGANIZATION; CYTOSKELETON_ORGANIZATION; CYTOSKELETON_DEPENDENT_INTRACELLULAR_TRANSPORT
TUBB	Beta-tubulin	upregulated in BC tissue [79]	PT	E2F_TARGETS	MICROTUBULE_BASED_PROCESS; CELL_DIVISION; CELL_CYCLE; CYTOSKELETON_ORGANIZATION; CYTOSKELETON_DEPENDENT_INTRACELLULAR_TRANSPORT; CELL_JUNCTION_ORGANIZATION
HSPE1/ CH10	Heat shock protein family E (HSP10) member/10kDa HSP	tumorigenesis [81], cancer cell survival, regulation of cell death [17]	PT	MYC_TARGETS_V1	PROTEIN_FOLDING/CMPF; PROGRAMMED_CELL_DEATH
				MTORC1-SIGNALING	
HSPD1	Heat shock protein family D (HSP60) member 1	cancer cell survival, regulation of cell death, proliferation [17], represses E-cadherin, promotes cell invasion, migration, poor prognosis [18]	PT	MYC_TARGETS_V1	BIOLOGICAL_ADHESION; PROTEIN_FOLDING; PROTEIN_REFOLDING; PROTEIN_MATURATION; PROTEIN_CONTAINING_COMPLEX_ORGANIZATION; PROTEIN_STABILIZATION; PROTEIN_INTRACELLULAR_TRANSPORT; TRANSMEMBRANE_TRANSPORT; PROTEIN_TRANSMEMBRANE_TRANSPORT_INTO_INTRACELLULAR_ORGANELLE; MITOCHONDRION_ORGANIZATION; DNA_RECOMBINATION; IMMUNE_RESPONSE_TO_TUMOR_CELL; PROGRAMMED_CELL_DEATH
				MTORC1_ SIGNALING	
				EMT [18]	
HSPA1A	Heat shock 70 kDa protein 1A variant	potential biomarker for BC, overexpressed in BC, promotes progression, inhibits apoptosis, extracellularly-activates proinflammatory immunity [22]	PT	COMPLEMENT	REGULATION_OF_CELL_DIFFERENTIATION; MITOTIC_SPINDLE_ORGANIZATION; MITOTIC_CELL_CYCLE_PROCESS; CYTOSKELETON_ORGANIZATION; PROTEIN_FOLDING/CMPF; PROTEIN_REFOLDING; PROTEIN_STABILIZATION; LYSOSOMAL_TRANSPORT; PROTEIN_CATABOLIC_PROCESS; RNA-CATABOLIC_PROCESS; MITOCHONDRION_ORGANIZATION; REGULATION_OF_DNA_TEMPLATED_TRANSCRIPTION_IN_RESPONSE_TO_STRESS PROGRAMMED_CELL_DEATH
TMSB10	Thymosin beta 10	overexpressed in many cancers: RCC, pancreatic, lung, and thyroid carcinoma, promotes migration, invasion, and EMT [82]; positively associated with high-grade aggressive BC, significantly elevated in BC cells and tissues, proliferation, invasion migration of BC cells by activation of Akt/FOXO signaling, valuable serum biomarker for diagnosis and potential therapeutic target in BC [83]	PT	EMT [83]	CYTOSKELETON_ORGANIZATION; CELL_MIGRATION
PARK7/DJ-1	Parkinsonism associated deglycase DJ-1	oncogene upregulated in various cancers, involved in tumor initiation, progression, proliferation, metastasis, recurrence, resistance to chemotherapy [84], overexpression increases cell survival; highly expressed in cytoplasm of invasive BC cells [85]	PT	-	RAS_PROTEIN_SIGNAL_TRANSDUCTION; INTRACELLULAR_PROTEIN_TRANSPORT; PROTEIN_MODIFICATION_BY_SMALL_PROTEIN_REMOVAL; PROTEIN_CATABOLIC_PROCES; PROTEIN_REPAIR; CELLULAR_AMINO_ACID_BIOSYNTHETIC_PROCESS; NUCLEOBASE_CONTAINING_SMALL_MOLECULE_METABOLIC_PROCESS; DNA_REPAIR; TRANSMEMBRANE_TRANSPORT; REGULATION_OF_SIGNALING_RECEPTOR_ACTIVITY; REGULATION_OF_TRANSCRIPTION_REGULATORY_REGION_DNA_BINDING; GENERATION_OF_PRECURSOR_METABOLITES_AND_ENERGY; MITOCHONDRION_ORGANIZATION; INFLAMMATORY_RESPONSE; PROGRAMMED_CELL_DEATH; CELL_DEATH_IN_RESPONSE_TO_HYDROGEN_PEROXIDE; DETOXIFICATION
PRDX2	Peroxiredoxin 2	overexpressed in various cancers [86], highly upregulated in BC [57]; dual effect in carcinogenesis, in BC induces selective growth of metastatic cancer cells in lung by protecting them against OS [87]; reduces OS, cell damage and apoptosis [88]	PT	PEROXIZOME	CELL_REDOX_HOMEOSTASIS
				ROS	
GAPDH1	Glyceraldehyde-3-phosphate dehydrogenase 1	overexpressed in many cancers [89], in association with BC cell proliferation and tumor aggressiveness [90]; several PTMs have pro-apoptotic role [73]; promotes cancer growth and metastasis by affecting EMT [91]	PT	GYCOLYSIS	CARBOHYDRATE_METABOLIC_PROCESS; nuclear tRNA export, DNA replication and repair, endocytosis, exocytosis, cytoskeletal organization, iron metabolism, cell death [89], membrane fusion, vesicle secretion, transcription co-activation, cell cycle regulation, mRNA stabilization [92]; DEFENSE_RESPONSE
				HYPOXIA	
				EMT [91]	
ACTC1	Actin alpha cardiac muscle 1	upregulated in BC and other malignancies [93]; promotes resistance to apoptosis, cell survival, controls cell migration [94]	PT	KRAS_SIGNALING_DN	Cell differentiation, anatomical structure development, cell cytoskeleton organization, programmed cell death
				APICAL_JUNCTION	
				EMT [95]	
PPIA/CYPA	Peptidylprolyl isomerase A	overexpressed in BC, cell survival [17], growth, malignant transformation, metastasis, drug resistance [38], anti-apoptosis [39]	PT	GLYCOLYSIS	CELL_ADHESION; CELL_MIGRATION; PROTEIN_FOLDING; PROTEIN_MODIFICATION_BY_SMALL_PROTEIN_CONJUGATION_OR_REMOVAL; CELL_DEATH_IN_RESPONSE_TO_OS
				MYC_TARGETS_V1	
Downregulated proteins and pathways					
ACTG1	Actin gamma 1	overexpressed in skin cancer and HCC, promotes growth, migration, proliferation, inhibits mitochondrial apoptotic pathway, increases aerobic glycolysis, role in microtubule integrity; depletion in BC cells resulted in centrosome amplification, formation of multipolar spindles, defects in chromosome segregation, leading to mitotic abnormalities [93]	AT	APICAL_JUNCTION	ACTOMYOSIN_STRUCTURE_ORGANIZATION; CYTOSKELETON_ORGANIZATION; CELL_MIGRATION; CELL_JUNCTION_ORGANIZATION; BIOLOGICAL_ADHESION
				CHOLESTEROL_HOMEOSTASIS	
TPI1	Triosephosphate isomerase	upregulated in multiple cancers, promotes tumor development and progression of BC in tissue and cell lines, promotes glycolysis, proliferation, metastasis, activates PI3K/Akt/mTOR, regulates EMT [55]	AT	GLYCOLYSIS	CARBOHYDRATE_METABOLIC_PROCESS
				MTORC1_SIGNALING	
				HYPOXIA	
HSPA1A	Heat shock 70kDa protein 1A variant	potential biomarker for BC, overexpressed in BC, promotes progression, inhibits apoptosis, extracellularly-activates proinflammatory immunity [22]	AT	COMPLEMENT	REGULATION_OF_CELL_DIFFERENTIATION; MITOTIC_SPINDLE_ORGANIZATION; MITOTIC_CELL_CYCLE_PROCESS/CELL_CYCLE_PROCESS; CYTOSKELETON_ORGANIZATION; PROTEIN_FOLDING/CMPF; PROTEIN_STABILIZATION; PROTEIN_CONTAINING_COMPLEX_ ORGANIZATION; LYSOSOMAL_TRANSPORT; PROTEIN_CATABOLIC_PROCESS; RNA-CATABOLIC_PROCESS; MITOCHONDRION_ORGANIZATION; REGULATION_OF_DNA_TEMPLATED_TRANSCRIPTION_IN_RESPONSE_TO_STRESS PROGRAMMED_CELL_DEATH
HSPB1	Heat shock 27 kDa protein 1	overexpressed in BC, downregulation was correlated with PTEN increase (tumor suppressor) that negatively regulates PI3K/AKT	AT	APOPTOSIS	PROTEIN_FOLDING/CMPF; CELL_ADHESION; CELL_MIGRATION; CYTOSKELETON_DEPENDENT_INTRACELLULAR_TRANSPORT; CELL_DEATH_IN_RESPONSE_TO_OXIDATIVE_STRESS; PROGRAMMED_CELL_DEATH
HSPA8	Heat shock 70 kDa protein 8	depletion suppresses cancer cells growth, induces apoptosis, and cell cycle arrest [96]	AT	G2M_CHECKPOINT	PROTEIN_FOLDING/CMPF; PROTEIN_REFOLDING;PROTEIN_CONTAINING_COMPLEX_ORGANIZATION; LYSOSOMAL_TRANSPORT; CMA & PROTEIN_CATABOLIC_PROCESS; CELL_JUNCTION_ORGANIZATION; CYTOSKELETON_DEPENDENT_INTRACELLULAR_TRANSPORT
FASN	Fatty acid synthase	inhibition reduces cell proliferation, suppresses migration and invasion and induces apoptosis [54]	AT	FAM	LIPID_BIOSYNTHETIC_PROCESS; FATTY_ACID_BIOSYNTHETIC_PROCESS; FATTY_ACID_METABOLIC_PROCESS
				CHOLESTEROL HOMEOSTASIS	
				ESTROGEN_RESPONSE_EARLY	
EEF1A1	Eukaryotic translation elongation factor-1 alpha-1	overexpressed in tumors, including BC, controls cell proliferation and cell death [46], promotes heat shock response, protecting cancer cells from proteotoxic stress, sustains cancer cell survival [47], oncogenesis, pro-apoptotic/anti-apoptotic activity	AT	-	CMA & PROTEIN_CATABOLIC_PROCESS; TRANSLATIONAL_ELONGATION
SOD1	Superoxide dismutase 1 (Cu-Zn)	downregulation promotes apoptosis and oncogene-induced senescence [61]	AT	ROS	CYTOSKELETON_ORGANIZATION; CELL_DEATH_IN_RESPONSE_TO_OXIDATIVE_STRESS; LIPID_METABOLIC_PROCESS; INFLAMMATORY_RESPONSE; PROGRAMMED_CELL_DEATH
				GLYCOLYSIS	
				PEROXIZOME	
				PROTEIN_SECRETION	
				APOPTOSIS	
MKI67	Proliferation marker protein Ki-67	overexpressed in cancer cells [97]; downregulated, reduces migration, invasion, tumor progression; knockout induces transcriptome remodeling, alters EMT and suppresses stem cell characteristics [98]	AT	G2M_CHECKPOINT	CHROMATIN_ORGANIZATION; CHROMOSOME_ORGANIZATION; CHROMOSOME_SEGREGATION; MITOTIC_NUCLEAR_DIVISION
CALM1	Calcium-calmodulin N-terminal domain	knockdown inhibits proliferation, invasion, migration, induces cell cycle arrest and increases apoptosis in ESCC [66]	AT	COMPLEMENT	CYTOSOLIC_CALCIUM_ION_TRANSPORT; TRANSMEMBRANE_TRANSPORT; CELL_CYCLE_PROCESS; MITOTIC_CELL_CYCLE; CYTOKINESIS
IFITM2	Interferon- induced transmembrane protein 2	downregulation inhibits migration and invasion in ccRCC [27]; knocking out IFITM2 enhanced activation of the endogenous IFN-α pathway that may alter the immune and stromal cells in TME enhancing the invasive abilities of cancer cells [99]	AT/ PT	IFN-α_RESPONSE	DEFENSE_RESPONSE; RESPONSE_TO_INTERFERON_ALPHA; RESPONSE_TO_INTERFERON_BETA; RESPONSE_TO_INTERFERON_GAMMA
				IFN-γ_RESPONSE	
RPS5	40S ribosomal protein S5	when overexpressed, negatively regulates the expression of p53 and plays an anti-apoptotic role in cancer cells [100]	AT	MYC_TARGETS_V1	CYTOPLASMIC_TRANSLATION; RIBOSOME_BIOGENESIS/RIBOSOME_ASSEMBLY
CTNNB1	Catenin beta 1	Nuclear CTNNB1 plays a key role in most cancers as an oncogene; downregulation inhibited cell proliferation, migration, and invasion (EMT) and induced apoptosis in RCC [101]	AT	WNT_BETA_CATENIN_SIGNALING	CELL_MORPHOGENESIS; BIOLOGICAL_ADHESION; CELL_CELL_JUNCTION_ASSEMBLY/CELL_JUNCTION_ORGANIZATION; REGULATION_OF_EPITHELIAL_TO_MESENCHYMAL_TRANSITION; CELL_MIGRATION; REGULATION_OF_DNA_TEMPLATED_TRANSCRIPTION_ELONGATION; RESPONSE_TO_ESTRADIOL; TELOMERE_ORGANIZATION; PROGRAMMED_CELL_DEATH
				CHOLESTEROL_HOMEOSTASIS	
				APOPTOSIS	
				TGF_BETA_SIGNALING	
				EMT [102]	
ISG15	Interferon-stimulated 15/Ubiquitin-like protein ISG15	putative oncogene, aberrantly expressed in human cancers, protumor/antitumor functions, overexpressed in highly metastatic MDA-MB-231 BC cell line, enhances proliferation or invasiveness [103], cell cycle progression, cell motility and tumor growth [104], overexpressed in BC tissue [105]	AT	IFN-α_RESPONSE	PROTEIN_CONTAINING_COMPLEX_ORGANIZATION; PROTEIN_MODIFICATION_BY_SMALL_PROTEIN_CONJUGATION; PROTEIN_MODIFICATION_BY_SMALL_PROTEIN_CONJUGATION_OR_REMOVAL; DEFENSE_RESPONSE, immune system modulation [105]
				IFN-γ_RESPONSE	
ANXA2	Annexin A	depletion resulted in ROS elevation upon OS and activation of ROS-mediated cellular damage/death, elevated protein oxidation, decreased tumor growth [106]	AT	HYPOXIA	PROTEIN_MATURATION; cellular redox regulation [106] MEMBRANE_ORGANIZATION; VESICLE_MEDIATED_TRANSPORT

AML-acute myeloid leukemia; AT-antitumorigenic; CC-cervical cancer; CMA-chaperone-mediated autophagy; CMPF-chaperone-mediated protein folding; EOC-epithelial ovarian cancer; ESCC-esophageal squamous cell carcinoma; EMT-epithelial-to-mesenchymal transition; ER-endoplasmic reticulum; ERAD-ER-associated degradation; FAM-fatty acid metabolism; GC-gastric cancer; GOBF-gene ontology biological process; HCC-hepatocellular carcinoma; HGSC-high-grade serous cancer; IFN-α-interferon alpha; IFN-γ-interferon gamma; LUAD-lung adenocarcinoma; MM-multiple myeloma; NSCLC-non-small cell lung cancer; OC-ovarian cancer; OXPHOS-oxidative phosphorylation; PCa-prostate cancer; PT-protumorigenic; RCC-renal cell carcinoma; ROS-REACTIVE_OXYGEN_SPECIES_PATHWAY; SKCM-skin cutaneous melanoma; UPR-unfolded protein response.

**Table 4:** Protumorigenic (in red) and antitumorigenic roles (in green) of JTB-related proteins.

	MYC_TARGETS_V1	MTORC1_SIGNALING	MITOTIC_SPINDLE	FAM	CHOLESTEROL_HOMEOSTASIS	COMPLEMENT	COAGULATION	UPR	TNFA_SIGNALING_VIA_NFKB	E2F_TARGETS	IF ALPHA	IF-GAMMA	EMT	PROTEIN_SECRETION	ERAD	GLYCOLYSIS	HYPOXIA	APOPTOSIS	OXPHOS	APICAL_JUNCTION	ESTOGEN_RESPONSE_EARLY	ANDROGEN_RESPONSE	PEROXIZOME	ROS	UV_RESPONSE_UP	WNT_BETA_CATENIN_SIGNALING	TGF_BETA_SIGNALING	KRAS_SIGNALING_DN	G2M_CHECKPOINT
**UP in UP**																													
HSPD1													[18]																
HSP90AA1		[107]											[21]																
HSPA1A													[23]																
RAN																													
RPL6																													
PPIA/CYPA													[40]																
ENO2																													
TUBA4A																													
TUBB2A																													
RPS14																													
PRKCSH																													
IFITM2																													
LAMP2																													
TPM3													[30]																
SLC25A5																		[45]											
**D in UP**																													
TPI1																													
FASN																													
CALM1																													
ACTN4																													
TPD52L2																													
SOD1																													
ENO1																													
YWHAQ																													
PRDX1																								[59]					
IQGAP2													[65]					[65]											
**UP in D**																													
HSPD1													[18]																
HSPE1																													
HSPA1A													[23]																
PPIA/CYPA													[40]																
PGK1													[108]																
ENO2																													
PSME1																													
GAPDH1													[91]																
PRDX2																													
TMSB10													[82]																
TUBA1A		[80]																											
TUBB																													
ACTC1													[95]																
PARK7																		[84]						[84]					
**D in D**																													
CTNNB1													[109]																
TPI1																													
FASN																													
SOD1																													
CALM1																													
ACTG1																													
HSPA1A													[23]																
HSPB1																													
HSPA8																													
ANXA2																								[106]					
ISG15																													
IFITM2																													
MKI67																													
RPS5																													

UP in UP JTB-upregulated proteins in overexpressed JTB condition; D in UP-downregulated proteins in overexpressed JTB condition; UP in D-upregulated protein in downregulated JTB condition; D in D-downregulated proteins in downregulated JTB condition.

## Data Availability

Raw data from Masslynx, HTML files from Mascot and Scaffold files will be provided upon request, according to Clarkson University Material Transfer Agreement.

## Supplementary Materials

The following are available online at https://www.mdpi.com/article/10.3390/molecules27238301/s1, Table S1. Proteins identified in our study. Table S2. Dysregulated proteins in our study.

## Sample Availability

Samples discussed in this manuscript (i.e., plasmids, protein products, cells and cell lines) will be provided upon request, according to Clarkson University Material Transfer Agreement.

## References

[1] Sung H., Ferlay J., Siegel R.L., Laversanne M., Soerjomataram I., Jemal A., Bray F. (2021). Global cancer statistics 2020: GLOBOCAN estimates of incidence and mortality worldwide for 36 cancers in 185 countries. CA A Cancer J. Clin..

[2] Wu L., Qu X. (2015). Cancer biomarker detection: Recent achievements and challenges. Chem. Soc. Rev..

[3] Chang J.Y.H., Ladame S., Ladame S., Chang J.Y.H. (2020). Chapter 1.1—Diagnostic, prognostic, and predictive biomarkers for cancer. Bioengineering Innovative Solutions for Cancer.

[4] Goossens N., Nakagawa S., Sun X., Hoshida Y. (2015). Cancer biomarker discovery and validation. Transl. Cancer Res..

[5] Strimbu K., Tavel J.A. (2010). What are biomarkers?. Curr. Opin. HIV AIDS.

[6] Spellman D.S., Deinhardt K., Darie C.C., Chao M.V., Neubert T.A. (2008). Stable isotopic labeling by amino acids in cultured primary neurons: Application to brain-derived neurotrophic factor-dependent phosphotyrosine-associated signaling. Mol. Cell. Proteom..

[7] Kanome T., Itoh N., Ishikawa F., Mori K., Kim-Kaneyama J., Nose K., Shibanuma M. (2007). Characterization of Jumping translocation breakpoint (JTB) gene product isolated as a TGF-β1-inducible clone involved in regulation of mitochondrial function, cell growth and cell death. Oncogene.

[8] Jayathirtha M., Channaveerappa D., Darie C. (2021). Investigation and Characterization of the Jumping Translocation Breakpoint (JTB) Protein using Mass Spectrometry based Proteomics. FASEB J..

[9] Xu X.F., Zhou S.W., Zhang X., Ye Z.Q., Zhang J.H., Ma X., Zheng T., Li H.Z. (2006). Prostate androgen-regulated gene: A novel potential target for androgen-independent prostate cancer therapy. Asian J. Androl..

[10] Hatakeyama S., Osawa M., Omine M., Ishikawa F. (1999). JTB: A novel membrane protein gene at 1q21 rearranged in a jumping translocation. Oncogene.

[11] Rousseau F., Pan B., Fairbrother W.J., Bazan J.F., Lingel A. (2012). The Structure of the Extracellular Domain of the Jumping Translocation Breakpoint Protein Reveals a Variation of the Midkine Fold. J. Mol. Biol..

[12] Platica O., Chen S., Ivan E., Lopingco M., Holland J., Platica M. (2000). PAR, a novel androgen regulated gene, ubiquitously expressed in normal and malignant cells. Int. J. Oncol..

[13] Jayathirtha M., Neagu A.-N., Whitham D., Alwine S., Darie C. (2022). Investigation of the effects of overexpression of jumping translocation breakpoint (JTB) protein in MCF7 cells for potential use as a biomarker in breast cancer. Am. J. Cancer Res..

[14] Jayathirtha M., Neagu A.-N., Whitham D., Alwine S., Darie C.C. (2022). Investigation of the effects of downregulation of jumping translocation breakpoint (JTB) protein expression in MCF7 cells for potential use as a biomarker in breast cancer. Am. J. Cancer Res..

[15] Aslebagh R., Channaveerappa D., Pentecost B.T., Arcaro K.F., Darie C.C. (2019). Combinatorial Electrophoresis and Mass Spectrometry-Based Proteomics in Breast Milk for Breast Cancer Biomarker Discovery. Adv. Exp. Med. Biol..

[16] Cox H.D., Chao C.-K., Patel S.A., Thompson C.M. (2008). Efficient digestion and mass spectral analysis of vesicular glutamate transporter 1: A recombinant membrane protein expressed in yeast. J. Proteome Res..

[17] Coumans J.V.F., Gau D., Poljak A., Wasinger V., Roy P., Moens P.D.J. (2014). Profilin-1 overexpression in MDA-MB-231 breast cancer cells is associated with alterations in proteomics biomarkers of cell proliferation, survival, and motility as revealed by global proteomics analyses. Omics.

[18] Kang B.-H., Shu C.-W., Chao J.-K., Lee C.-H., Fu T.-Y., Liou H.-H., Ger L.-P., Liu P.-F. (2019). HSPD1 repressed E-cadherin expression to promote cell invasion and migration for poor prognosis in oral squamous cell carcinoma. Sci. Rep..

[19] Liu H., Zhang Z., Huang Y., Wei W., Ning S., Li J., Liang X., Liu K., Zhang L. (2021). Plasma HSP90AA1 Predicts the Risk of Breast Cancer Onset and Distant Metastasis. Front. Cell Dev. Biol..

[20] Condelli V., Crispo F., Pietrafesa M., Lettini G., Matassa D.S., Esposito F., Landriscina M., Maddalena F. (2019). HSP90 Molecular Chaperones, Metabolic Rewiring, and Epigenetics: Impact on Tumor Progression and Perspective for Anticancer Therapy. Cells.

[21] Liu K., Chen J., Yang F., Zhou Z., Liu Y., Guo Y., Hu H., Gao H., Li H., Zhou W. (2019). BJ-B11, an Hsp90 Inhibitor, Constrains the Proliferation and Invasion of Breast Cancer Cells. Front. Oncol..

[22] de Freitas G.B., Penteado L., Miranda M.M., Filassi J.R., Baracat E.C., Linhares I.M. (2022). The circulating 70kDa heat shock protein (HSPA1A) level is a potential biomarker for breast carcinoma and its progression. Sci. Rep..

[23] Nikotina A.D., Vladimirova S.A., Komarova E.Y., Alexeev D., Efremov S., Leonova E., Pavlov R., Kartsev V.G., Polonik S.G., Margulis B.A. (2021). Prevention of High Glucose-Mediated EMT by Inhibition of Hsp70 Chaperone. Int. J. Mol. Sci..

[24] Sheng C., Qiu J., Wang Y., He Z., Wang H., Wang Q., Huang Y., Zhu L., Shi F., Chen Y. (2018). Knockdown of Ran GTPase expression inhibits the proliferation and migration of breast cancer cells. Mol. Med. Rep..

[25] Wang X., Yao S., Luo G., Zhou Y., Fang Q. (2021). Downregulation of RPS14 inhibits the proliferation and metastasis of estrogen receptor-positive breast cancer cells. Anti-Cancer Drugs.

[26] Wu Q., Gou Y., Wang Q., Jin H., Cui L., Zhang Y., He L., Wang J., Nie Y., Shi Y. (2011). Downregulation of RPL6 by siRNA inhibits proliferation and cell cycle progression of human gastric cancer cell lines. PLoS ONE.

[27] Yang N., Liu Z., Pang S., Wu J., Liang J., Sun L. (2021). Predicative value of IFITM2 in renal clear cell carcinoma: IFITM2 is associated with lymphatic metastasis and poor clinical outcome. Biochem. Biophys. Res. Commun..

[28] Yao B., Qu S., Hu R., Gao W., Jin S., Ju J., Zhao Q. (2019). Delivery of platelet TPM3 mRNA into breast cancer cells via microvesicles enhances metastasis. FEBS Open Bio.

[29] Chen S., Shen Z., Gao L., Yu S., Zhang P., Han Z., Kang M. (2021). TPM3 mediates epithelial-mesenchymal transition in esophageal cancer via MMP2/MMP9. Ann. Transl. Med..

[30] Choi H.-S., Yim S.-H., Xu H.-D., Jung S.-H., Shin S.-H., Hu H.-J., Jung C.-K., Choi J.Y., Chung Y.-J. (2010). Tropomyosin3 overexpression and a potential link to epithelial-mesenchymal transition in human hepatocellular carcinoma. BMC Cancer.

[31] Gao S., Wang S., Zhao Z., Zhang C., Liu Z., Ye P., Xu Z., Yi B., Jiao K., Naik G.A. (2022). TUBB4A interacts with MYH9 to protect the nucleus during cell migration and promotes prostate cancer via GSK3β/β-catenin signalling. Nat. Commun..

[32] Patsialou A., Wang Y., Lin J., Whitney K., Goswami S., Kenny P., Condeelis J. (2012). Selective gene-expression profiling of migratory tumor cells in vivo predicts clinical outcome in breast cancer patients. Breast Cancer Res. BCR.

[33] Kallergi G., Aggouraki D., Zacharopoulou N., Stournaras C., Georgoulias V., Martin S.S. (2018). Evaluation of α-tubulin, detyrosinated α-tubulin, and vimentin in CTCs: Identification of the interaction between CTCs and blood cells through cytoskeletal elements. Breast Cancer Res..

[34] Shin D., Park J., Han D., Moon J.H., Ryu H.S., Kim Y. (2020). Identification of TUBB2A by quantitative proteomic analysis as a novel biomarker for the prediction of distant metastatic breast cancer. Clin. Proteom..

[35] Saha T. (2012). LAMP2A overexpression in breast tumors promotes cancer cell survival via chaperone-mediated autophagy. Autophagy.

[36] Hao Y., Kacal M., Ouchida A.T., Zhang B., Norberg E., Vakifahmetoglu-Norberg H. (2019). Targetome analysis of chaperone-mediated autophagy in cancer cells. Autophagy.

[37] Shin G.-C., Moon S.U., Kang H.S., Choi H.-S., Han H.D., Kim K.-H. (2019). PRKCSH contributes to tumorigenesis by selective boosting of IRE1 signaling pathway. Nat. Commun..

[38] Nigro P., Pompilio G., Capogrossi M.C. (2013). Cyclophilin A: A key player for human disease. Cell Death Dis..

[39] Cheng S., Luo M., Ding C., Peng C., Lv Z., Tong R., Xiao H., Xie H., Zhou L., Wu J. (2016). Downregulation of Peptidylprolyl isomerase A promotes cell death and enhances doxorubicin-induced apoptosis in hepatocellular carcinoma. Gene.

[40] Guo Y., Jiang M., Zhao X., Gu M., Wang Z., Xu S., Yue W. (2018). Cyclophilin A promotes non-small cell lung cancer metastasis via p38 MAPK. Thorac. Cancer.

[41] Liu C.-c., Dsaa A., Wang W., Wang L., Liu W.-j., Wang J.-h., Geng Q.-r., Lu Y. (2018). ENO2 Promotes Cell Proliferation, Glycolysis, and Glucocorticoid-Resistance in Acute Lymphoblastic Leukemia. Cell. Physiol. Biochem..

[42] Liu D., Mao Y., Chen C., Zhu F., Lu W., Ma H. (2020). Expression patterns and clinical significances of ENO2 in lung cancer: An analysis based on Oncomine database. Ann. Transl. Med..

[43] Gómez-Cebrián N., Domingo-Ortí I., Poveda J.L., Vicent M.J., Puchades-Carrasco L., Pineda-Lucena A. (2021). Multi-Omic Approaches to Breast Cancer Metabolic Phenotyping: Applications in Diagnosis, Prognosis, and the Development of Novel Treatments. Cancers.

[44] Lytovchenko O., Kunji E.R.S. (2017). Expression and putative role of mitochondrial transport proteins in cancer. Biochim. Biophys. Acta (BBA)-Bioenerg..

[45] Jang J.-Y., Choi Y., Jeon Y.-K., Kim C.-W. (2008). Suppression of adenine nucleotide translocase-2 by vector-based siRNA in human breast cancer cells induces apoptosis and inhibits tumor growth in vitro and in vivo. Breast Cancer Res..

[46] Abbas W., Kumar A., Herbein G. (2015). The eEF1A Proteins: At the Crossroads of Oncogenesis, Apoptosis, and Viral Infections. Front. Oncol..

[47] Lin C.-Y., Beattie A., Baradaran B., Dray E., Duijf P.H.G. (2018). Contradictory mRNA and protein misexpression of EEF1A1 in ductal breast carcinoma due to cell cycle regulation and cellular stress. Sci. Rep..

[48] Eigentler A., Tymoszuk P., Zwick J., Schmitz A.A., Pircher A., Kocher F., Schlicker A., Lesche R., Schäfer G., Theurl I. (2020). The Impact of Cand1 in Prostate Cancer. Cancers.

[49] Alhammad R. (2022). Bioinformatics Identification of TUBB as Potential Prognostic Biomarker for Worse Prognosis in ERα-Positive and Better Prognosis in ERα-Negative Breast Cancer. Diagnostics.

[50] López-Mateo I., Villaronga M.Á., Llanos S., Belandia B. (2012). The transcription factor CREBZF is a novel positive regulator of p53. Cell Cycle.

[51] Fang J., Jiang G., Mao W., Huang L., Huang C., Wang S., Xue H., Ke J., Ni Q. (2022). Up-regulation of long noncoding RNA MBNL1-AS1 suppresses breast cancer progression by modulating miR-423-5p/CREBZF axis. Bioengineered.

[52] Luo X., Cheng C., Tan Z., Li N., Tang M., Yang L., Cao Y. (2017). Emerging roles of lipid metabolism in cancer metastasis. Mol. Cancer.

[53] Xu S., Chen T., Dong L., Li T., Xue H., Gao B., Ding X., Wang H., Li H. (2020). Fatty acid synthase promotes breast cancer metastasis by mediating changes in fatty acid metabolism. Oncol. Lett..

[54] Zhang W., Huang J., Tang Y., Yang Y., Huaidong H. (2020). Inhibition of Fatty Acid Synthase (FASN) Affects the Proliferation and Apoptosis of HepG2 Hepatoma Carcinoma Cells via the β-catenin/C-myc Signaling Pathway. Ann. Hepatol..

[55] Jin X., Wang D., Lei M., Guo Y., Cui Y., Chen F., Sun W., Chen X. (2022). TPI1 activates the PI3K/AKT/mTOR signaling pathway to induce breast cancer progression by stabilizing CDCA5. J. Transl. Med..

[56] Ding C., Fan X., Wu G. (2017). Peroxiredoxin 1—An antioxidant enzyme in cancer. J. Cell. Mol. Med..

[57] Bajor M., Zych A.O., Graczyk-Jarzynka A., Muchowicz A., Firczuk M., Trzeciak L., Gaj P., Domagala A., Siernicka M., Zagozdzon A. (2018). Targeting peroxiredoxin 1 impairs growth of breast cancer cells and potently sensitises these cells to prooxidant agents. Br. J. Cancer.

[58] Guo Q.J., Mills J.N., Bandurraga S.G., Nogueira L.M., Mason N.J., Camp E.R., Larue A.C., Turner D.P., Findlay V.J. (2013). MicroRNA-510 promotes cell and tumor growth by targeting peroxiredoxin1 in breast cancer. Breast Cancer Res. BCR.

[59] Jezierska-Drutel A., Attaran S., Hopkins B.L., Skoko J.J., Rosenzweig S.A., Neumann C.A. (2019). The peroxidase PRDX1 inhibits the activated phenotype in mammary fibroblasts through regulating c-Jun N-terminal kinases. BMC Cancer.

[60] Powell L.E., Foster P.A. (2021). Protein disulphide isomerase inhibition as a potential cancer therapeutic strategy. Cancer Med..

[61] Gomez M.L., Shah N., Kenny T.C., Jenkins E.C., Germain D. (2019). SOD1 is essential for oncogene-driven mammary tumor formation but dispensable for normal development and proliferation. Oncogene.

[62] Cancemi P., Buttacavoli M., Roz E., Feo S. (2019). Expression of Alpha-Enolase (ENO1), Myc Promoter-Binding Protein-1 (MBP-1) and Matrix Metalloproteinases (MMP-2 and MMP-9) Reflect the Nature and Aggressiveness of Breast Tumors. Int. J. Mol. Sci..

[63] Zhang Y., Yang D., Wei Z., Zhang X., Hu Z., Fu H., Xu J., Wang W. (2022). The Antitumor Effect of TPD52L2 Silencing on Oxaliplatin-Resistant Gastric Carcinoma Is Related to Endoplasmic Reticulum Stress In Vitro. Evid.-Based Complement. Altern. Med. Ecam.

[64] Hsu K.-S., Kao H.-Y. (2013). Alpha-actinin 4 and tumorigenesis of breast cancer. Vitam. Horm..

[65] Kumar D., Patel S.A., Hassan M.K., Mohapatra N., Pattanaik N., Dixit M. (2021). Reduced IQGAP2 expression promotes EMT and inhibits apoptosis by modulating the MEK-ERK and p38 signaling in breast cancer irrespective of ER status. Cell Death Dis..

[66] Liu T., Han X., Zheng S., Liu Q., Tuerxun A., Zhang Q., Yang L., Lu X. (2021). CALM1 promotes progression and dampens chemosensitivity to EGFR inhibitor in esophageal squamous cell carcinoma. Cancer Cell Int..

[67] Ochieng J., Nangami G., Sakwe A., Moye C., Alvarez J., Whalen D., Thomas P., Lammers P. (2018). Impact of Fetuin-A (AHSG) on Tumor Progression and Type 2 Diabetes. Int. J. Mol. Sci..

[68] Zheng Y., Zhou Z., Wei R., Xiao C., Zhang H., Fan T., Zheng B., Li C., He J. (2022). The RNA-binding protein PCBP1 represses lung adenocarcinoma progression by stabilizing DKK1 mRNA and subsequently downregulating β-catenin. J. Transl. Med..

[69] Zhang J., Wang K., Zhang J., Liu S.S., Dai L., Zhang J.-Y. (2011). Using proteomic approach to identify tumor-associated proteins as biomarkers in human esophageal squamous cell carcinoma. J. Proteome Res..

[70] Sanchez-Martin D., Martinez-Torrecuadrada J., Teesalu T., Sugahara K., Alvarez de Cienfuegos A., Ximénez-Embún P., Fernández-Periáñez R., Martín M., Molina-Privado I., Ruppen I. (2013). Proteasome activator complex PA28 identified as an accessible target in prostate cancer by in vivo selection of human antibodies. Proc. Natl. Acad. Sci. USA.

[71] Wang Q., Pan F., Li S., Huang R., Wang X., Wang S., Liao X., Li D., Zhang L. (2019). The prognostic value of the proteasome activator subunit gene family in skin cutaneous melanoma. J. Cancer.

[72] Gu Y., Barwick B.G., Shanmugam M., Hofmeister C.C., Kaufman J., Nooka A., Gupta V., Dhodapkar M., Boise L.H., Lonial S. (2020). Downregulation of PA28α induces proteasome remodeling and results in resistance to proteasome inhibitors in multiple myeloma. Blood Cancer J..

[73] Ananthi S., Lakshmi N., Paul A., Kumaraswamy A., Mahalingam S. (2018). Global Quantitative Proteomics reveal Deregulation of Cytoskeletal and Apoptotic Signalling Proteins in Oral Tongue Squamous Cell Carcinoma. Sci. Rep..

[74] Zhang D., Lim S.G., Koay E.S.-C. (2007). Proteomic identification of down-regulation of oncoprotein DJ-1 and proteasome activator subunit 1 in hepatitis B virus-infected well-differentiated hepatocellular carcinoma. Int. J. Oncol..

[75] Fu D., He C., Wei J., Zhang Z., Luo Y., Tan H., Ren C. (2018). PGK1 is a Potential Survival Biomarker and Invasion Promoter by Regulating the HIF-1α–Mediated Epithelial-Mesenchymal Transition Process in Breast Cancer. Cell. Physiol. Biochem..

[76] Husi H., Skipworth R.J.E., Cronshaw A., Fearon K.C.H., Ross J.A. (2016). Proteomic identification of potential cancer markers in human urine using subtractive analysis. Int. J. Oncol..

[77] Barger C.J., Zhang W., Sharma A., Chee L., James S.R., Kufel C.N., Miller A., Meza J., Drapkin R., Odunsi K. (2018). Expression of the POTE gene family in human ovarian cancer. Sci. Rep..

[78] Hsu M.-T., Wang Y.-K., Tseng Y.J. (2022). Exosomal Proteins and Lipids as Potential Biomarkers for Lung Cancer Diagnosis, Prognosis, and Treatment. Cancers.

[79] Nami B., Wang Z. (2018). Genetics and Expression Profile of the Tubulin Gene Superfamily in Breast Cancer Subtypes and Its Relation to Taxane Resistance. Cancers.

[80] Wang D., Jiao Z., Ji Y., Zhang S. (2020). Elevated TUBA1A Might Indicate the Clinical Outcomes of Patients with Gastric Cancer, Being Associated with the Infiltration of Macrophages in the Tumor Immune Microenvironment. J. Gastrointest. Liver Dis..

[81] Zoppino F.C.M., Guerrero-Gimenez M.E., Castro G.N., Ciocca D.R. (2018). Comprehensive transcriptomic analysis of heat shock proteins in the molecular subtypes of human breast cancer. BMC Cancer.

[82] Xiao R., Shen S., Yu Y., Pan Q., Kuang R., Huang H. (2019). TMSB10 promotes migration and invasion of cancer cells and is a novel prognostic marker for renal cell carcinoma. Int. J. Clin. Exp. Pathol..

[83] Zhang X., Ren D., Guo L., Wang L., Wu S., Lin C., Ye L., Zhu J., Li J., Song L. (2017). Thymosin beta 10 is a key regulator of tumorigenesis and metastasis and a novel serum marker in breast cancer. Breast Cancer Res. BCR.

[84] Jin W. (2020). Novel Insights into PARK7 (DJ-1), a Potential Anti-Cancer Therapeutic Target, and Implications for Cancer Progression. J.Clin. Med..

[85] Kim R.H., Peters M., Jang Y., Shi W., Pintilie M., Fletcher G.C., DeLuca C., Liepa J., Zhou L., Snow B. (2005). DJ-1, a novel regulator of the tumor suppressor PTEN. Cancer Cell.

[86] Wang W., Wei J., Zhang H., Zheng X., Zhou H., Luo Y., Yang J., Deng Q., Huang S., Fu Z. (2021). PRDX2 promotes the proliferation of colorectal cancer cells by increasing the ubiquitinated degradation of p53. Cell Death Dis..

[87] Nicolussi A., D’Inzeo S., Capalbo C., Giannini G., Coppa A. (2017). The role of peroxiredoxins in cancer. Mol. Clin. Oncol..

[88] Li H., Yang H., Wang D., Zhang L., Ma T. (2020). Peroxiredoxin2 (Prdx2) Reduces Oxidative Stress and Apoptosis of Myocardial Cells Induced by Acute Myocardial Infarction by Inhibiting the TLR4/Nuclear Factor kappa B (NF-κB) Signaling Pathway. Med. Sci. Monit..

[89] Zhang J.-Y., Zhang F., Hong C.-Q., Giuliano A.E., Cui X.-J., Zhou G.-J., Zhang G.-J., Cui Y.-K. (2015). Critical protein GAPDH and its regulatory mechanisms in cancer cells. Cancer Biol. Med..

[90] Révillion F., Pawlowski V., Hornez L., Peyrat J. (2000). Glyceraldhyde-3-phosphate dehydrogenase gene expression in human breast cancer. Eur. J. Cancer.

[91] Liu K., Tang Z., Huang A., Chen P., Liu P., Yang J., Lu W., Liao J., Sun Y., Wen S. (2016). Glyceraldehyde-3-phosphate dehydrogenase promotes cancer growth and metastasis through upregulation of SNAIL expression. Int. J. Oncol..

[92] Tarrado-Castellarnau M., Diaz-Moralli S., Polat I.H., Sanz-Pamplona R., Alenda C., Moreno V., Castells A., Cascante M. (2017). Glyceraldehyde-3-phosphate dehydrogenase is overexpressed in colorectal cancer onset. Transl. Med. Commun..

[93] Suresh R., Diaz R.J. (2021). The remodelling of actin composition as a hallmark of cancer. Transl. Oncol..

[94] Suresh R., Picard D., Lo R., Beaulieu J., Remke M., Diaz R.J. (2021). Expression of cell type incongruent alpha-cardiac actin 1 subunit in medulloblastoma reveals a novel mechanism for cancer cell survival and control of migration. Neuro-Oncol. Adv..

[95] Xin H.-W., Ambe C.M., Miller T.C., Chen J.-Q., Wiegand G.W., Anderson A.J., Ray S., Mullinax J.E., Hari D.M., Koizumi T. (2016). Liver Label Retaining Cancer Cells Are Relatively Resistant to the Reported Anti-Cancer Stem Cell Drug Metformin. J. Cancer.

[96] Li J., Ge Z. (2021). High HSPA8 expression predicts adverse outcomes of acute myeloid leukemia. BMC Cancer.

[97] Wu S.-Y., Liao P., Yan L.-Y., Zhao Q.-Y., Xie Z.-Y., Dong J., Sun H.-T. (2021). Correlation of MKI67 with prognosis, immune infiltration, and T cell exhaustion in hepatocellular carcinoma. BMC Gastroenterol..

[98] Mrouj K., Andrés-Sánchez N., Dubra G., Singh P., Sobecki M., Chahar D., Al Ghoul E., Aznar A.B., Prieto S., Pirot N. (2021). Ki-67 regulates global gene expression and promotes sequential stages of carcinogenesis. Proc. Natl. Acad. Sci. USA.

[99] Shi Y., Du L., Lv D., Li H., Shang J., Lu J., Zhou L., Bai L., Tang H. (2019). Exosomal Interferon-Induced Transmembrane Protein 2 Transmitted to Dendritic Cells Inhibits Interferon Alpha Pathway Activation and Blocks Anti-Hepatitis B Virus Efficacy of Exogenous Interferon Alpha. Hepatology.

[100] Watanabe M., Boku S., Kobayashi K., Kurumida Y., Sukeno M., Masuda M., Mizushima K., Kato C., Iizumi Y., Hirota K. (2022). A chemoproteoinformatics approach demonstrates that aspirin increases sensitivity to MEK inhibition by directly binding to RPS5. PNAS Nexus.

[101] Yang C.-M., Ji S., Li Y., Fu L.-Y., Jiang T., Meng F.-D. (2017). β-Catenin promotes cell proliferation, migration, and invasion but induces apoptosis in renal cell carcinoma. Onco Targets Ther..

[102] Kim W.K., Kwon Y., Jang M., Park M., Kim J., Cho S., Jang D.G., Lee W.-B., Jung S.H., Choi H.J. (2019). β-catenin activation down-regulates cell-cell junction-related genes and induces epithelial-to-mesenchymal transition in colorectal cancers. Sci. Rep..

[103] Desai S.D. (2015). ISG15: A double edged sword in cancer. Oncoimmunology.

[104] Bolado-Carrancio A., Lee M., Ewing A., Muir M., Macleod K.G., Gallagher W.M., Nguyen L.K., Carragher N.O., Semple C.A., Brunton V.G. (2021). ISGylation drives basal breast tumour progression by promoting EGFR recycling and Akt signalling. Oncogene.

[105] Bektas N., Noetzel E., Veeck J., Press M.F., Kristiansen G., Naami A., Hartmann A., Dimmler A., Beckmann M.W., Knüchel R. (2008). The ubiquitin-like molecule interferon-stimulated gene 15 (ISG15) is a potential prognostic marker in human breast cancer. Breast Cancer Res..

[106] Madureira P.A., Hill R., Miller V.A., Giacomantonio C.A., Lee P.W.K., Waisman D.M. (2011). Annexin A2 is a novel Cellular Redox Regulatory Protein involved in Tumorigenesis. Oncotarget.

[107] Yan L.-R., Shen S.-X., Wang A., Xi D., Liu Y.-N., Yuan Y., Xu Q. (2021). Comprehensive Pan-Cancer Analysis of Heat Shock Protein 110, 90, 70, and 60 Families. Front. Mol. Biosci..

[108] Zhang Y., Cai H., Liao Y., Zhu Y., Wang F., Hou J. (2020). Activation of PGK1 under hypoxic conditions promotes glycolysis and increases stem cell-like properties and the epithelial-mesenchymal transition in oral squamous cell carcinoma cells via the AKT signalling pathway. Int. J. Oncol..

[109] Conacci-Sorrell M., Zhurinsky J., Ben-Ze’ev A. (2002). The cadherin-catenin adhesion system in signaling and cancer. J. Clin. Investig..

[110] Leggett S.E., Hruska A.M., Guo M., Wong I.Y. (2021). The epithelial-mesenchymal transition and the cytoskeleton in bioengineered systems. Cell Commun. Signal..

[111] González-Mariscal L., Miranda J., Gallego-Gutiérrez H., Cano-Cortina M., Amaya E. (2020). Relationship between apical junction proteins, gene expression and cancer. Biochim. Biophys. Acta (BBA)-Biomembr..

[112] Gehren A.S., Rocha M.R., de Souza W.F., Morgado-Díaz J.A. (2015). Alterations of the apical junctional complex and actin cytoskeleton and their role in colorectal cancer progression. Tissue Barriers.

[113] Xiao L., Peng H., Yan M., Chen S. (2021). Silencing ACTG1 Expression Induces Prostate Cancer Epithelial Mesenchymal Transition Through MAPK/ERK Signaling Pathway. DNA Cell Biol..

[114] Tentler D., Lomert E., Novitskaya K., Barlev N.A. (2019). Role of ACTN4 in Tumorigenesis, Metastasis, and EMT. Cells.

[115] Honda K. (2015). The biological role of actinin-4 (ACTN4) in malignant phenotypes of cancer. Cell Biosci..

[116] Tanabe S., Kawabata T., Aoyagi K., Yokozaki H., Sasaki H. (2016). Gene expression and pathway analysis of CTNNB1 in cancer and stem cells. World J. Stem Cells.

[117] Ferreira L.T., Figueiredo A.C., Orr B., Lopes D., Maiato H. (2018). Dissecting the role of the tubulin code in mitosis. Methods Cell Biol..

[118] Zhang L., Fan M., Napolitano F., Gao X., Xu Y., Li L. (2021). Transcriptomic analysis identifies organ-specific metastasis genes and pathways across different primary sites. J. Transl. Med..

[119] Calderwood S. (2018). Heat shock proteins and cancer: Intracellular chaperones or extracellular signalling ligands?. Philos. Trans. R. Soc. B Biol. Sci..

[120] Wang Y., Dong C., Zhou B.P. (2020). Metabolic reprogram associated with epithelial-mesenchymal transition in tumor progression and metastasis. Genes Dis..

[121] Kondaveeti Y., Guttilla Reed I.K., White B.A. (2015). Epithelial–mesenchymal transition induces similar metabolic alterations in two independent breast cancer cell lines. Cancer Lett..

[122] Luu T. (2021). Epithelial-Mesenchymal Transition and Its Regulation Mechanisms in Pancreatic Cancer. Front. Oncol..

[123] Rebane-Klemm E., Truu L., Reinsalu L., Puurand M., Shevchuk I., Chekulayev V., Timohhina N., Tepp K., Bogovskaja J., Afanasjev V. (2020). Mitochondrial Respiration in KRAS and BRAF Mutated Colorectal Tumors and Polyps. Cancers.

[124] Gill K.S., Fernandes P., O’Donovan T.R., McKenna S.L., Doddakula K.K., Power D.G., Soden D.M., Forde P.F. (2016). Glycolysis inhibition as a cancer treatment and its role in an anti-tumour immune response. Biochim. Biophys. Acta (BBA)-Rev. Cancer.

[125] Zhang H., Zhang D., Hu X. (2022). A Potential Fatty Acid Metabolism-Related Gene Signature for Prognosis in Clear Cell Renal Cell Carcinoma. Cancers.

[126] Hare S.H., Harvey A.J. (2017). mTOR function and therapeutic targeting in breast cancer. Am. J. Cancer Res..

[127] Paplomata E., O’Regan R. (2014). The PI3K/AKT/mTOR pathway in breast cancer: Targets, trials and biomarkers. Ther. Adv. Med. Oncol..

[128] Koundouros N., Poulogiannis G. (2020). Reprogramming of fatty acid metabolism in cancer. Br. J. Cancer.

[129] Cheng C.-S., Wang Z., Chen J. (2014). Targeting FASN in Breast Cancer and the Discovery of Promising Inhibitors from Natural Products Derived from Traditional Chinese Medicine. Evid.-Based Complement. Altern. Med..

[130] Rossato F.A., Zecchin K.G., La Guardia P.G., Ortega R.M., Alberici L.C., Costa R.A.P., Catharino R.R., Graner E., Castilho R.F., Vercesi A.E. (2014). Fatty acid synthase inhibitors induce apoptosis in non-tumorigenic melan-a cells associated with inhibition of mitochondrial respiration. PLoS ONE.

[131] Röhrig F., Schulze A. (2016). The multifaceted roles of fatty acid synthesis in cancer. Nat. Rev. Cancer.

[132] Zhu S., Wang W., Zhang J., Ji S., Jing Z., Chen Y.Q. (2022). Slc25a5 regulates adipogenesis by modulating ERK signaling in OP9 cells. Cell. Mol. Biol. Lett..

[133] Bartholomeusz C., Gonzalez-Angulo A.M., Liu P., Hayashi N., Lluch A., Ferrer-Lozano J., Hortobágyi G.N. (2012). High ERK protein expression levels correlate with shorter survival in triple-negative breast cancer patients. Oncologist.

[134] Dong Z., Cui H. (2018). The Autophagy-Lysosomal Pathways and Their Emerging Roles in Modulating Proteostasis in Tumors. Cells.

[135] Rodvold J.J., Chiu K.T., Hiramatsu N., Nussbacher J.K., Galimberti V., Mahadevan N.R., Willert K., Lin J.H., Zanetti M. (2017). Intercellular transmission of the unfolded protein response promotes survival and drug resistance in cancer cells. Sci. Signal..

[136] Sannino S., Brodsky J.L. (2017). Targeting protein quality control pathways in breast cancer. BMC Biol..

[137] Santamaría P.G., Mazón M.J., Eraso P., Portillo F. (2019). UPR: An Upstream Signal to EMT Induction in Cancer. J. Clin. Med..

[138] Arias E., Cuervo A.M. (2020). Pros and Cons of Chaperone-Mediated Autophagy in Cancer Biology. Trends Endocrinol. Metab..

[139] Kaushik S., Cuervo A.M. (2012). Chaperone-mediated autophagy: A unique way to enter the lysosome world. Trends Cell Biol..

[140] Joung E.K., Kim J., Yoon N., Maeng L.-S., Kim J.H., Park S., Kang K., Kim J.S., Ahn Y.-H., Ko Y.H. (2019). Expression of EEF1A1 Is Associated with Prognosis of Patients with Colon Adenocarcinoma. J. Clin. Med..

[141] Falvey C.M., O’Donovan T.R., El-Mashed S., Nyhan M.J., O’Reilly S., McKenna S.L. (2017). UBE2L6/UBCH8 and ISG15 attenuate autophagy in esophageal cancer cells. Oncotarget.

[142] Nagaraj N.S., Singh O.V., Merchant N.B. (2010). Proteomics: A strategy to understand the novel targets in protein misfolding and cancer therapy. Expert Rev. Proteom..

[143] Kim S.-K., Kim K., Ryu J.-W., Ryu T.-Y., Lim J.H., Oh J.-H., Min J.-K., Jung C.-R., Hamamoto R., Son M.-Y. (2019). The novel prognostic marker, EHMT2, is involved in cell proliferation via HSPD1 regulation in breast cancer. Int. J. Oncol..

[144] Elhamamsy A.R., Metge B.J., Alsheikh H.A., Shevde L.A., Samant R.S. (2022). Ribosome Biogenesis: A Central Player in Cancer Metastasis and Therapeutic Resistance. Cancer Res..

[145] Pecoraro A., Pagano M., Russo G., Russo A. (2021). Ribosome Biogenesis and Cancer: Overview on Ribosomal Proteins. Int. J. Mol. Sci..

[146] Vizirianakis I.S., Papachristou E.T., Andreadis P., Zopounidou E., Matragkou C.N., Tsiftsoglou A.S. (2015). Genetic manipulation of RPS5 gene expression modulates the initiation of commitment of MEL cells to erythroid maturation: Implications in understanding ribosomopathies. Int. J. Oncol..

[147] Sánchez-Aragó M., Formentini L., Cuezva J.M. (2013). Mitochondria-mediated energy adaption in cancer: The H(+)-ATP synthase-geared switch of metabolism in human tumors. Antioxid. Redox Signal..

[148] Polson E.S., Kuchler V.B., Abbosh C., Ross E.M., Mathew R.K., Beard H.A., da Silva B., Holding A.N., Ballereau S., Chuntharpursat-Bon E. (2018). KHS101 disrupts energy metabolism in human glioblastoma cells and reduces tumor growth in mice. Sci. Transl. Med..

[149] Galai G., Ben-David H., Levin L., Orth M.F., Grünewald T.G.P., Pilosof S., Berstein S., Rotblat B. (2020). Pan-Cancer Analysis of Mitochondria Chaperone-Client Co-Expression Reveals Chaperone Functional Partitioning. Cancers.

[150] Leu J.I.J., Barnoud T., Zhang G., Tian T., Wei Z., Herlyn M., Murphy M.E., George D.L. (2017). Inhibition of stress-inducible HSP70 impairs mitochondrial proteostasis and function. Oncotarget.

[151] Chen Y., Yang S., Zhou H., Su D. (2020). PRDX2 Promotes the Proliferation and Metastasis of Non-Small Cell Lung Cancer In Vitro and In Vivo. BioMed Res. Int..

[152] Yuan L., Cai Y., Zhang L., Liu S., Li P., Li X. (2022). Promoting Apoptosis, a Promising Way to Treat Breast Cancer With Natural Products: A Comprehensive Review. Front. Pharmacol..

[153] Lin S., Zhang Y.-J. (2017). Interference of Apoptosis by Hepatitis B Virus. Viruses.

[154] Liu Y.-P., Yang X.-N., Jazag A., Pan J.-S., Hu T.-H., Liu J.-J., Guleng B., Ren J.-L. (2012). HBsAg inhibits the translocation of JTB into mitochondria in HepG2 cells and potentially plays a role in HCC progression. PLoS ONE.

[155] Kennedy D., Jäger R., Mosser D.D., Samali A. (2014). Regulation of apoptosis by heat shock proteins. IUBMB Life.

[156] Heimes A.-S., Härtner F., Almstedt K., Krajnak S., Lebrecht A., Battista M.J., Edlund K., Brenner W., Hasenburg A., Sahin U. (2020). Prognostic Significance of Interferon-γ and Its Signaling Pathway in Early Breast Cancer Depends on the Molecular Subtypes. Int. J. Mol. Sci..

[157] Provance O.K., Lewis-Wambi J. (2019). Deciphering the role of interferon alpha signaling and microenvironment crosstalk in inflammatory breast cancer. Breast Cancer Res..

[158] Tecalco-Cruz A.C., Macías-Silva M., Ramírez-Jarquín J.O., Méndez-Ambrosio B. (2021). Identification of genes modulated by interferon gamma in breast cancer cells. Biochem. Biophys. Rep..

[159] Jorgovanovic D., Song M., Wang L., Zhang Y. (2020). Roles of IFN-γ in tumor progression and regression: A review. Biomark. Res..

[160] Karnoub A.E., Dash A.B., Vo A.P., Sullivan A., Brooks M.W., Bell G.W., Richardson A.L., Polyak K., Tubo R., Weinberg R.A. (2007). Mesenchymal stem cells within tumour stroma promote breast cancer metastasis. Nature.

[161] Desai S., Reed R., Burks J., Wood L., Pullikuth A., Haas A., Liu L., Breslin J., Meiners S., Sankar S. (2011). ISG15 disrupts cytoskeletal architecture and promotes motility in human breast cancer cells. Exp. Biol. Med..

[162] Andersen J.B., Hassel B.A. (2006). The interferon regulated ubiquitin-like protein, ISG15, in tumorigenesis: Friend or foe?. Cytokine Growth Factor Rev..

[163] Dupree E.J., Manzoor Z., Alwine S., Crimmins B.S., Holsen T.M., Darie C.C. (2022). Proteomic analysis of the lake trout (Salvelinus namaycush) heart and blood: The beginning of a comprehensive lake trout protein database. Proteomics.

[164] Mihăşan M., Babii C., Aslebagh R., Channaveerappa D., Dupree E., Darie C.C. (2018). Proteomics based analysis of the nicotine catabolism in Paenarthrobacter nicotinovorans pAO1. Sci. Rep..

[165] Sokolowska I., Dorobantu C., Woods A.G., Macovei A., Branza-Nichita N., Darie C.C. (2012). Proteomic analysis of plasma membranes isolated from undifferentiated and differentiated HepaRG cells. Proteome Sci..

[166] Channaveerappa D., Lux J.C., Wormwood K.L., Heintz T.A., McLerie M., Treat J.A., King H., Alnasser D., Goodrow R.J., Ballard G. (2017). Atrial electrophysiological and molecular remodelling induced by obstructive sleep apnoea. J. Cell. Mol. Med..

